# Extracting time series matching a small-angle X-ray scattering profile from trajectories of molecular dynamics simulations

**DOI:** 10.1038/s41598-022-13982-9

**Published:** 2022-06-15

**Authors:** Masahiro Shimizu, Aya Okuda, Ken Morishima, Rintaro Inoue, Nobuhiro Sato, Yasuhiro Yunoki, Reiko Urade, Masaaki Sugiyama

**Affiliations:** Institute for Integrated Radiation and Nuclear Science, Kyoto University, Kumatori, Sennan-gun, Osaka, 590-0494 Japan

**Keywords:** Computational biophysics, Biophysics, Nanoscale biophysics

## Abstract

Solving structural ensembles of flexible biomolecules is a challenging research area. Here, we propose a method to obtain possible structural ensembles of a biomolecule based on small-angle X-ray scattering (SAXS) and molecular dynamics simulations. Our idea is to clip a time series that matches a SAXS profile from a simulation trajectory. To examine its practicability, we applied our idea to a multi-domain protein ER-60 and successfully extracted time series longer than 1 micro second from trajectories of coarse-grained molecular dynamics simulations. In the extracted time series, the domain conformation was distributed continuously and smoothly in a conformational space. Preferred domain conformations were also observed. Diversity among scattering curves calculated from each ER-60 structure was interpreted to reflect an open-close motion of the protein. Although our approach did not provide a unique solution for the structural ensemble of the biomolecule, each extracted time series can be an element of the real behavior of ER-60. Considering its low computational cost, our approach will play a key role to identify biomolecular dynamics by integrating SAXS, simulations, and other experiments.

## Introduction

Many proteins are composed of multiple domains that realize complex and sophisticated functions by rearranging their domains flexibly. For example, each domain works together to introduce DNA supercoiling in DNA topoisomerases and each domain cooperates to refold substrate proteins in some foldases^1–4^. However, revealing their dynamic processes in solution remains challenging.

Small-angle X-ray scattering (SAXS) is a representative experimental technique to study such behavior of biomolecules^5–9^. The SAXS profiles include structural information in the range of approximately 1–100 nm, which is usually wide enough to study structural range of multi-domain proteins or their complexes. SAXS intensity I(**Q**) is calculated as a function of scattering vector (**Q**):

1 $$ {\text{Q}} = 4{\uppi }\sin {\uptheta }/{\lambda  } $$

2 $$ {\text{I}}\left( {\mathbf{Q}} \right) = \mathop \sum \limits_{{{\text{i}} = 1}}^{{\text{N}}} \mathop \sum \limits_{{{\text{j}} = 1}}^{{\text{N}}} {\text{f}}_{{\text{i}}} \left( {\mathbf{Q}} \right){\text{f}}_{{\text{j}}} \left( {\mathbf{Q}} \right)\frac{{\sin \left( {{\mathbf{Q}}{\text{r}}_{{{\text{ij}}}} } \right)}}{{{\mathbf{Q}}{\text{r}}_{{{\text{ij}}}} }} $$

In Eq. (1), the θ and λ are the scattering angle and the wavelength of the incident X-ray, respectively. Equation (2) is termed Debye’s equation^9^: N is the number of atoms in a system, r_ij_ is the distance between the i-th and j-th atoms, and f_i_ (**Q**) is an atomic form factor of i-th atom, which is regarded as a constant in the measured small-angle range.

Since all molecules in a sample solution contribute an experimental SAXS profile, the profile includes information on their structural ensemble. There could be two different types of structural sets of which ensembles reproduce the same SAXS profile. One is a “homogeneous structural set” in which all molecules have a similar structure. The other is a “heterogeneous structural set”. The latter includes the diverse structures, but their averaged SAXS profile reproduces the experimental one. It is difficult to determine which set should be adopted as the state of the molecule in solution. In other words, we cannot judge whether all molecules have similar or diverse structures only from an experimental SAXS profile.

To address this issue, two main criteria are considered in the context of structural modeling; they are reviewed as “maximum parsimony” and “maximum entropy”^10^. In the “maximum parsimony” approach, a structural set composed of a small number of models is selected among possible structural sets matching an experimental SAXS profile. For example, the Akaike information criterion or Bayesian information criterion is calculated for possible structural sets, and a structural set minimizing the criteria is chosen^11,12^. Many algorithms, such as ensemble optimization, minimal ensemble search, sparse ensemble selection, and maximum occurrence, have been proposed and utilized^13–17^.

In the “maximum entropy” approach, a free energy landscape derived from a simulation force field is adopted as the prior distribution. The resultant ensemble should match an experimental SAXS profile and be least inconsistent with the force field. A force field does not always reproduce a real structural ensemble of a molecule. Therefore, correction of a free energy landscape to match an experimental SAXS profiles is often effective. There are two ways to correct a free energy landscape while satisfying entropy maximization. One direct approach is reweighting the free energy landscape to match an experimental SAXS profile after structural sampling by molecular dynamics (MD) simulations or Monte Carlo simulations^18–22^. The other approach is to perform parallel simulations with additional potential to reproduce an experimental SAXS profile^23–27^.

Although both methods are effective, they are not necessarily sufficient to model any biomolecular systems. The structural set composed of a small number of models implies that the resulting structures discretely distribute in a structural space. In a largely fluctuating system, such as a multi-domain protein with intrinsic disordered regions, it is more reasonable to model a structural set which continuously distributes in a structure space.

Maximum entropy approaches are useful in that they can construct a physically reasonable structural set. It is necessary to sufficiently explore possible molecular structures in these approaches. However, sufficient stuructural sampling is often difficult for atomistic MD simulations. Coarse-grained (CG) MD simulation is a useful alternative to overcome this difficulty^19,26^. In many CG models, each CG bead reproduces net charge and hydrophilicity of their corresponding atom set, resulting in roughly reasonable inter- and intra-molecular interfaces in the simulations^28–30^. However, parameter adjustment using experimental data is often required^31,32^. A general-purpose CGMD potential does not necessarily guarantee accurate dissociation constant for the interfaces of a biomolecule. Therefore, it may not always be suitable to perform entropy maximization using a free energy landscape from a given CGMD potential as the prior distribution.

Here, we propose another approach to enumerate possible behaviors of a biomolecule using an experimental SAXS profile and CGMD simulations. In this method, CGMD simulations are first performed to obtain trajectories that efficiently cover their conformational space. Then, time series that match the SAXS profile are extracted from the trajectories. If the resulting time series are long enough, they reflect information on possible preferred states of the biomolecule; the molecule stays in the stable states for a longer time than unstable ones.

**Figure 1:**
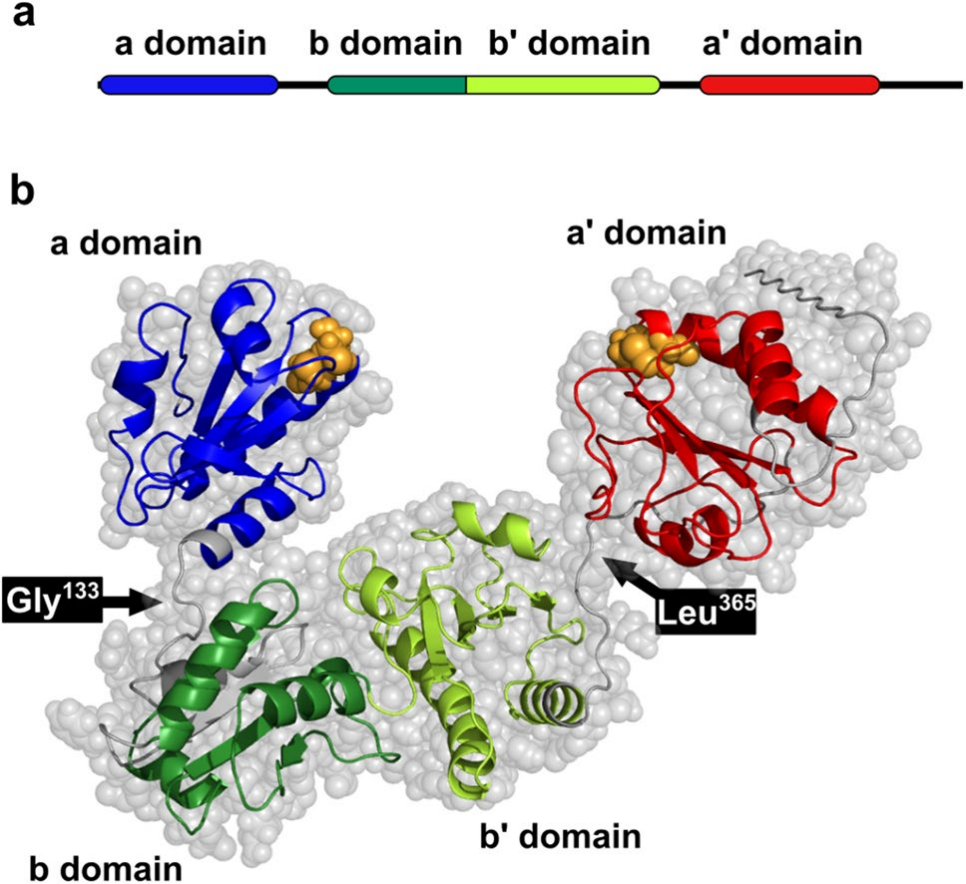
Multi-domain protein ER-60. (**a**) Four domains constituting ER-60. (**b**) Structural model of ER-60 based on a crystal structure (PDBID: 3F8U)^33^. PyMOL^39^ was used to model the missing C-terminal residues and replace the mutated C60A with cysteine. In the ribbon model, the **a** domain in shown in blue, the **b** domain in green, the **b′** domain in yellowish green, and the **a′** domain in red. The other parts are gray. The reactive cysteines in the CGHC motif are orange. Gly^133^ and Leu^365^ are hinge regions between the **a** and the **b** domain, and the **b′** and the **a′** domain, respectively.

We tested our method on a multi-domain protein ER-60, which is a member of protein disulfide isomerase family^3^. ER-60 is composed **a**, **b**, **b′**, **a′** domains and possesses reaction Cys-Gly-His-Cys (CGHC) motifs in both the **a** and **a′** domains (Fig. 1). The **a** and **a′** domains are respectively connected to the **b** and **b′** domains via short hinge regions^3,33^. We focused on the domain dynamics of ER-60. We could extract multiple time series with our method. By examining domain conformation of ER-60 in each of the time series, we got overview of possible structural ensembles of the multi-domain protein. Distance between the **a** and **a′** domains almost linearly correlated with I(Q) at each Q value. Therefore, diversity in scattering curves among structures was explained by open-close motion of ER-60. In addition, this linear relationship was indicated as two isosbestic points in Q-I(Q) plot. The actual structural ensemble of ER-60 in solution can be a mixture of these possible domain dynamics. Our method provides "elements of biomolecular motion”, which should be useful in the context of an integrated structural biology including SAXS, MD simulations, and other experiments.

## Methods

### Modeling strategy

To compose a series of structural models that reproduces a SAXS profile, we made the following two assumptions about CGMD:

1. When stable inter- or intra- protein interfaces appear in CGMD simulations, they are regarded as candidates of actual interfaces, and
2. Since atomistic-scale interactions, such as hydrogen bonds and hydrophobic interactions, cannot be expressed precisely in CGMD simulations, the accurate affinity of the interfaces is not guaranteed.

Based on these assumptions, we devised a method to collect a series of structural models that include possible stable states. This is composed of two steps. In step 1, CGMD simulations changing a possible parameter are performed. In step 2, from each of the trajectories, the longest continuous time series that reproduces the SAXS profile is extracted. In summary, we clip a part of a CGMD trajectory that matches a SAXS profile and approximates the actual behavior of the biomolecule as a repetition of the clipped time series. We designate this method “SAS-CLIP”. Although only a single region is clipped from each trajectory, we can expect that possible structural ensembles can be enumerated by applying the SAS-CLIP to multiple trajectories. This point is studied in the “Results and discussion” section below. Using the multi-domain protein ER-60 as a model system, we examined feasibility and investigated the resulting structural series of SAS-CLIP.

### MD simulations

All simulations were performed using GROMACS 2020.4^34,35^. Before CGMD simulations, it was necessary to clarify the structural stability of three folded regions, from Ser^25^ to Pro^134^ (system **a**), that from Ala^132^ to Lys^366^ (system **b-b′**), and from Tyr^364^ to Glu^493^ (system **a′**). For this purpose, single atomistic simulations for the three systems were first performed.

CGMD simulations were performed using the Martini 3 open-beta version. In the CGMD simulations, the Lennard–Jones potential between water and ER-60 was scaled, as in previous reports^19,20^. The Lennard–Jones potential between i-th and j-th particles is described as:

$$ {\text{E}}_{{{\text{Lennard}} - {\text{Jones}}}} = 4{\upvarepsilon }_{{{\text{ij}}}} \left[ {\left( {\frac{{{\upsigma }_{{{\text{ij}}}} }}{{{\text{r}}_{{{\text{ij}}}} }}} \right)^{12} - \left( {\frac{{{\upsigma }_{{{\text{ij}}}} }}{{{\text{r}}_{{{\text{ij}}}} }}} \right)^{6} } \right] $$

In this study, we regarded the ε_ij_ as a parameter. When i-th and j-th bead pair is water-protein bead pair, the ε_ij_ is treated as λ_WP_ ε_ij,default_. Here, the ε_ij,default_ is the default value in the Martini 3 open-beta version, and λ_WP_ is the scaling factor for water-protein interactions. The σ_ij_s are constants depending on bead type, and r_ij_ is distance between i-th and j-th particles. First, we performed three 5000-ns production runs with λ_WP_ values of 1.0, 1.01, 1.02, 1.03, 1.04, 1.05, and 1.06. According to the results (described in “Results and discussion” section), we additionally performed two 5000-ns production runs with λ_WP_ of 1.035, 1.043, 1.045, 1.046, 1.049, 1.052, and 1.055. We also performed two 10,000-ns production runs with λ_WP_ values of 1.04, 1.043, 1.046, 1.049, 1.052, and 1.055. Snapshots taken every 2 ns were used for analysis. For simulation detail, please see the Supplementary Information.

### Analysis

SAXS profiles of snapshots in the CGMD simulations were calculated by Pepsi-SAXS^36^. Since the Pepsi-SAXS requires an atomistic model, the CGMD snapshots were reverse-mapped with the software backward^37^ before running the Pepsi-SAXS. Detailed description of the reverse-mapping is in the Supplementary Information.

The χ^2^ value is given as follows:

$$ {\upchi }^{2} = \frac{1}{{{\text{N}} - 1}}\mathop \sum \limits_{{{\text{i}} = 1}}^{{\text{N}}} \left( {\frac{{{\text{I}}_{{{\text{exp}}}} \left( {{\text{Q}}_{{\text{i}}} } \right) - {\text{I}}_{{{\text{sim}}}} \left( {{\text{Q}}_{{\text{i}}} } \right)}}{{{\upsigma }\left( {{\text{Q}}_{{\text{i}}} } \right)}}} \right)^{2} $$

$$ {\text{I}}_{{{\text{sim}}}} \left( {{\text{Q}}_{{\text{i}}} } \right) = {\text{c}}\mathop \sum \limits_{{{\text{j}} = 1}}^{{\text{M}}} {\text{I}}_{{{\text{sim}},{\text{ j}}}} \left( {{\text{Q}}_{{\text{i}}} } \right) - {\text{offs}} $$

where N numbers of data points and M is the number of models in a structure set. I_exp_(q_i_) and I_sim,j_ (q_i_) are the scattering intensities of experimental SAXS profile and calculated scattering intensity of j th model, respectively. The theoretical profiles are normalized by I_sim,j_(0). c and offs are adjustment parameters. The latter parameter explains possible mismatch between buffer solution and sample solution in the experimental SAXS profile. The parameters are determined by the condition: $\frac{{\partial {\upchi }^{2} }}{{\partial {\text{c}}}} = 0$ and $\frac{{\partial {\upchi }^{2} }}{{\partial {\text{offs}}}} = 0$.

The distance distribution function p (r) was calculated using the reverse-mapped atomistic model. For the calculation, all electrons were considered to be in the center of an atom, and hydrogen atoms were ignored.

Since snapshots were acquired every 2 ns for analysis, the duration time of the time series L_t_ was calculated by multiplying the number of snapshots in the time series by 2 ns/frame.

The structure of ER-60 was analyzed based on the domain positions and orientations. Domains of ER-60 are mainly defined based on domain database Pfam^38^, where ER-60 consists of three domains: from Ser^26^ to Lys^130^ (**a** domain), from Phe^160^ to Asp^355^ (**b-b′** domains), and from Pro^377^ to Arg^482^ (**a′** domain). In previous reports, ER-60 was treated as four domain protein^3,4^, where the **b-b′** domain was further divided into **b** and **b′** domains. The θ_a-b-b′_, θ_b-b′-a′_, and φ_a-b-b′-a′_ are angles formed by centers of mass (COMs) of the three domains or dihedral angle formed by COMs of the four domains (Supplementary Fig. S1). φ_b′-b-a-CGHC(a)_ and φ_b-b′-a′-CGHC(a′)_ are angles formed by COMs of the three domains and the COM of the CGHC motif of either the **a** or **a′** domain. The D_**a-a**′_ is distance between COMs of **a** and **a′** domains. The COM of each domain or CGHC motif is defined as the averaged coordinates of backbone beads of the corresponding residues (Supplementary Fig. S1). Probability distributions were calculated by binning a variable space and counting the number of data points in each bin.

The difference in the shape of the (θ_a-b-b′_, θ_b-b′-a′_) probability map between a clipped time series with the SAS-CLIP and its original trajectory (i.e., the entire trajectory before performing the SAS-CLIP) was evaluated using the following Kullback–Leibler divergence:

$$ {\text{KL}}_{{{\text{SAS}} - {\text{CLIP}}}} = \mathop \sum \limits_{{\left( {{\uptheta }_{{{\text{a}} - {\text{b}} - {\text{b}}^{\prime}}} ,{{\uptheta }}_{{{\text{b}} - {\text{b}}^{\prime} - {\text{a}}^{\prime}}} } \right) \subset {\mathbf{C}}1 \cup {\mathbf{C}}2}} {\text{P}}_{{{\text{SAS}} - {\text{CLIP}}}} \left( {{\uptheta }_{{{\text{a}} - {\text{b}} - {\text{b}}^{\prime} }} ,{{\uptheta }}_{{{\text{b}} - {\text{b}}^{\prime} - {\text{a}}^{\prime} }} } \right)\log \frac{{{\text{P}}_{{{\text{SAS}} - {\text{CLIP}}}} \left( {{\uptheta }_{{{\text{a}} - {\text{b}} - {\text{b}}^{\prime} }} ,{{\uptheta }}_{{{\text{b}} - {\text{b}}^{\prime} - {\text{a}}^{\prime} }} } \right)}}{{{\text{P}}_{{{\text{original}}}} \left( {{\uptheta }_{{{\text{a}} - {\text{b}} - {\text{b}}^{\prime} }} ,{{\uptheta }}_{{{\text{b}} - {\text{b}}^{\prime} - {\text{a}}^{\prime} }} } \right)}} $$

Here, P_SAS-CLIP_ (θ_a-b-b′_, θ_b-b′-a′_) is the probability at (θ_a-b-b′_, θ_b-b′-a′_) of a clipped time series, and P_original_ (θ_a-b-b′_, θ_b-b′-a′_) is the probability of the original trajectory. KL_SAS-CLIP_ only considers the region where P_SAS-CLIP_ (θ_a-b-b′_, θ_b-b′-a′_) > 0 and their vicinity. In other words, the sum was calculated only for ${\mathbf{C}}1 \cup {\mathbf{C}}2$, where **C1** and **C2** are defined as follows (a schematic is presented in Supplementary Fig. S1d):

**C1**. {(θ_a-b-b′_, θ_b-b′-a′_) | P_SAS-CLIP_ (θ_a-b-b′_, θ_b-b′-a′_) > 0}

**C2**. {(θ_a-b-b′_, θ_b-b′-a′_) | Adjacent to **C1**}These conditions allow KL_SAS-CLIP_ to be small when ER-60 stays only in a few (or one) of several stable states. Considering that the KL_SAS-CLIP_ ignores outside the ${\mathbf{C}}1 \cup {\mathbf{C}}2$, the P_original_ (θ_a-b-b′_, θ_b-b′-a′_) is normalized to meet the following relationship:

$$ \mathop \sum \limits_{{\left( {{\uptheta }_{{{\text{a}} - {\text{b}} - {\text{b}}\prime }} ,{{\uptheta}}_{{{\text{b}} - {\text{b}}\prime - {\text{a}}\prime }} } \right) \subset {\mathbf{C}}1 \cup {\mathbf{C}}2}} {\text{P}}_{{{\text{original}}}} \left( {{\uptheta }_{{{\text{a}} - {\text{b}} - {\text{b}}\prime }} ,{{\uptheta}}_{{{\text{b}} - {\text{b}}\prime - {\text{a}}\prime }} } \right) = 1 $$

Graphs and figures were created using gnuplot and inkscape. Images of protein structures were created using PyMOL^39^.

## Results and discussion

### Origin of differences between crystal and solution structures of ER-60

Our previous study showed that the structure of ER-60 in solution differs from the crystal structure^4^ because the SAXS profile calculated from crystal structure did not reproduce the experimental structure. To clarify the origin of this discrepancy, we first examined the dynamics of each folded structure. We performed atomistic MD simulations of three parts of ER-60, including the **a**, **b-b′**, and **a′** domains. In any of the three simulations, root mean square deviation (RMSD) of the folded region between a simulation snapshot and the crystal structure distributed at approximately 1.5 Å (Supplementary Figs. S2, S3, S4). In the **a**-, **b-b′**-, and **a′**-part simulations, the prevalence of simulation snapshot with RMSD > 2.0 Å were 12.8%, 0.25%, and 21.2%, respectively (Supplementary Fig. S4; all supplementary data are provided). These findings suggest that the structure of each four domain of ER-60 in solution is almost the same as the crystal structure. Therefore, the discrepancy between crystal and solution structures should originate from the domain conformation or domain dynamics in solution.

### Feasibility of SAS-CLIP

**Figure 2:**
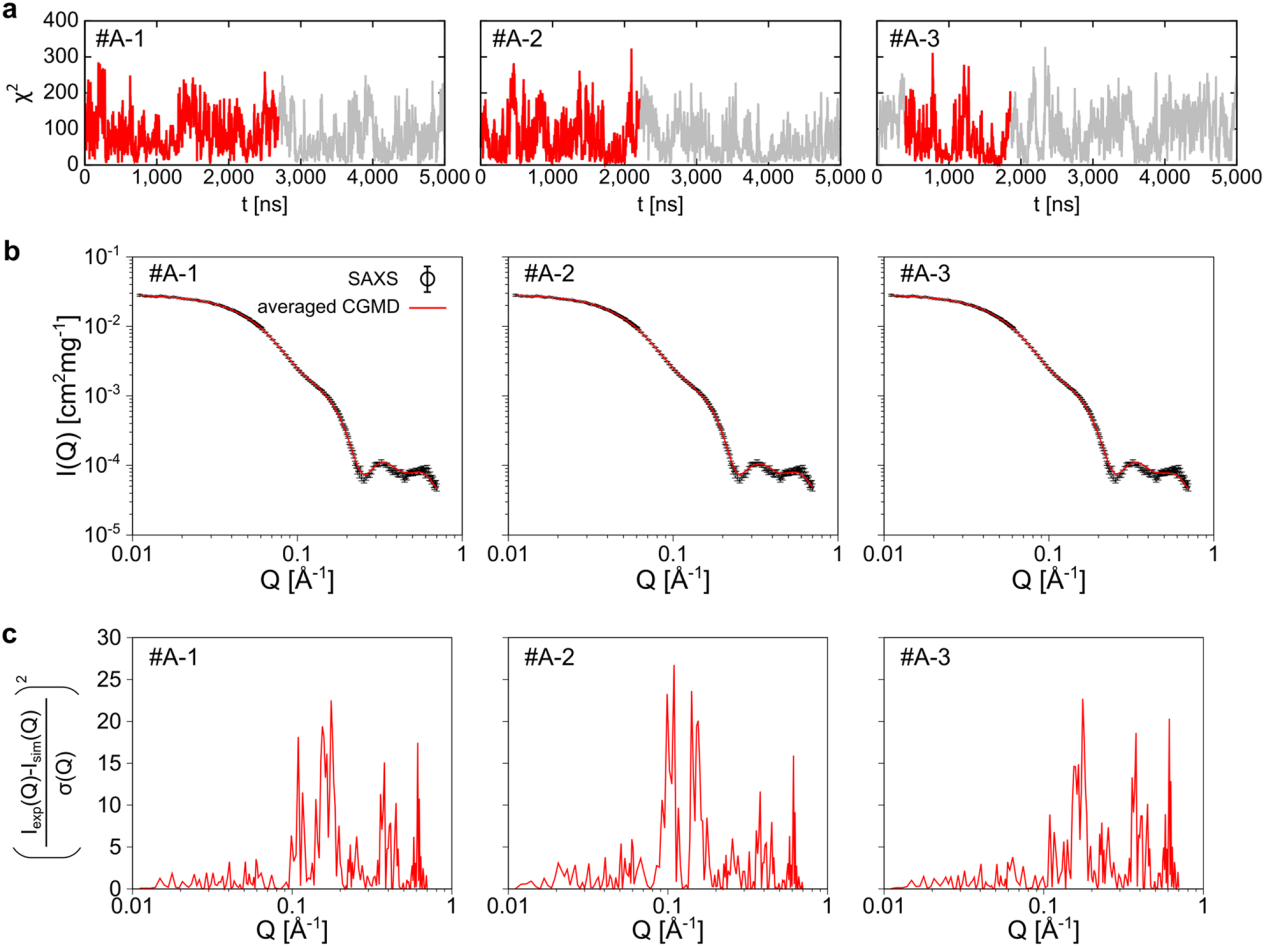
Extracting time series satisfying χ^2^ < 3.0 from CGMD simulation trajectories by the SAS-CLIP. (**a**) Trajectories of χ^2^ during CGMD simulations. The regions corresponding to the clipped time series #A-1, #A-2, and #A-3 are shown in red. The other regions are shown in gray. (**b**) Averaged SAXS profiles of the time series #A-1, #A-2 #A-3 (red lines) are compared with the experimental SAXS profile (black circle with error bars of s.d.). (**c**) The squared residuals of the averaged scattering intensities.

First, we examined whether a time series with a small χ^2^ could be clipped with SAS-CLIP. Three 5000-ns CGMD simulations were performed for each condition of λ_WP_ = 1.0, 1.01, 1.02, 1.03, 1.04, 1.05, and 1.06. The SAS-CLIP was applied to each trajectory with “χ^2^ < 3.0” as the criterion for reproducing an experimental SAXS profile which we previously reported^4^. Figure 2 shows the three longest time series, #A-1, #A-2, and #A-3 provided by the SAS-CLIP. Each averaged scattering curve matched the experimental one, suggesting that SAS-CLIP worked well (Fig. 2b). The duration time (L_t_) of #A-1, #A-2, and #A-3 were 2690, 2208, and 1462 ns, respectively (Table 1). The times were long enough to elucidate several preferred conformations originating from the CGMD force field (Supplementary Fig. S5). The finding indicated that SAS-CLIP can capture physically reasonable structural series. Interestingly, χ^2^ values for each simulation snapshot in the series were distributed broadly in the range below 350 (Supplementary Fig. S6). Nevertheless, the entire structure series reproduced the experimental SAXS profile. These structural sets could not be obtained by simply collecting individual structures with small χ^2^.

**Table 1:** λ_WP_, L_t_, χ^2^, and KL_SAS-CLIP_ of time series obtained by the SAS-CLIP.

ID	Criteria	λ_WP_	L_t_ [ns]	χ^2^	KL_SAS-CLIP_	
#A-1	χ^2^ < 3.0	1.04	2690	2.99	0.120	
#A-2		1.04	2208	2.98	0.163	
#A-3		1.06	1462	3.00	0.421	
#B-1	I. χ^2^ < 3.0 II. squared residuals < 12.5 (Q < 0.25)	1.045	1572	2.19	0.418	
#B-2		1.04	1372	2.32	0.340	Same trajectory as #A-1
#B-3		1.04	1338	2.03	0.266	Same trajectory as #A-2
#B-4		1.055	1268	2.56	0.287	
#B-5		1.046	1146	2.59	0.543	
#B-6		1.052	1076	2.45	0.659	10-μs simulation

### Criteria for a time series to be consistent with both CGMD simulations and a SAXS profile

**Figure 3:**
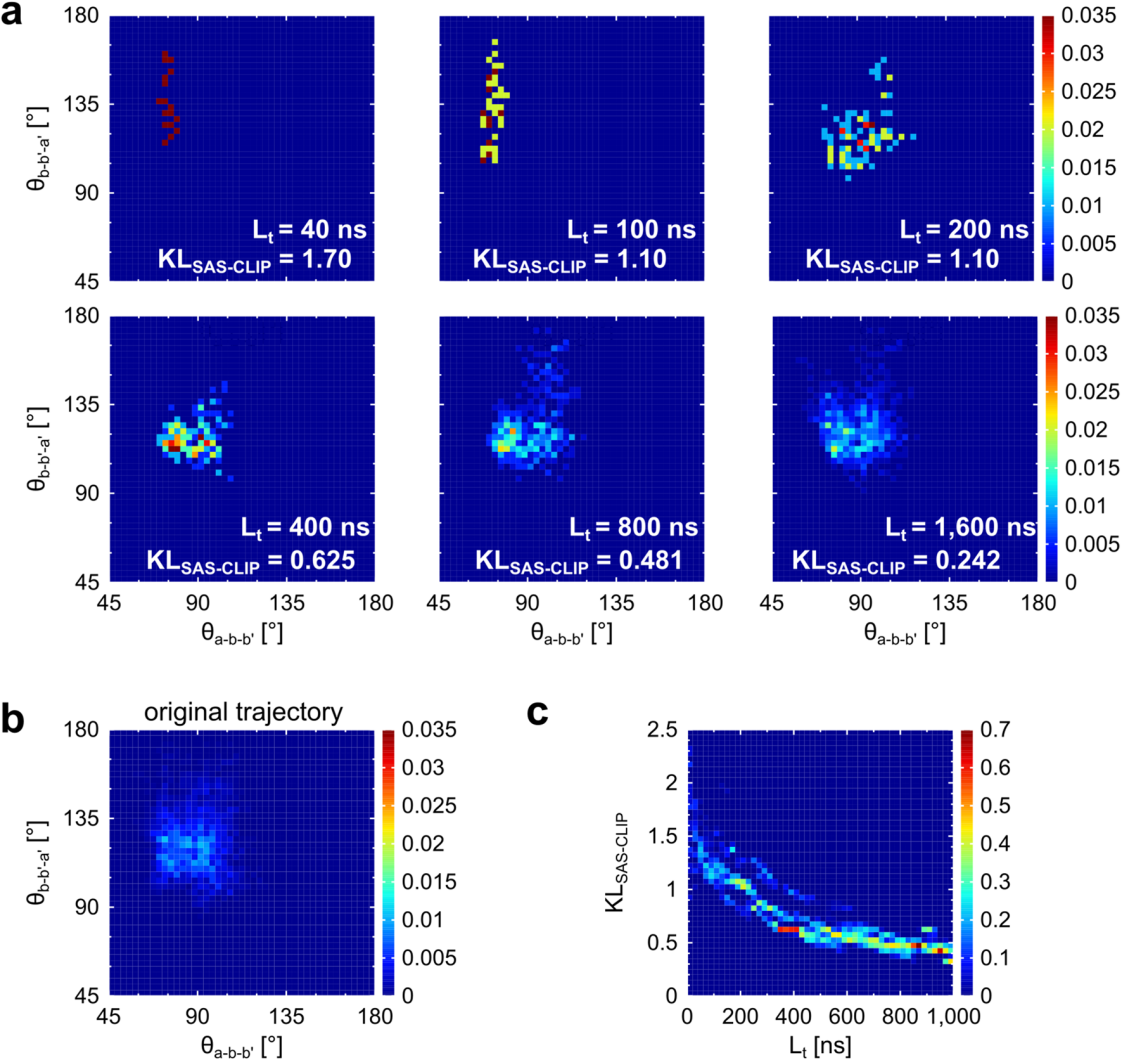
Relationship between L_t_ and KL_SAS-CLIP_. (**a**) Domain conformations of several clipped time series with various L_t_. These were clipped from the same CGMD trajectory as the #A-2. All satisfy χ^2^ < 3.0. The colors in the heatmaps show the appearance probability in the (θ_a-b-b′_, θ_b-b′-a′_) space. The pixel with the probability > 0.035 is presented in the same color as that with the probability of 0.035. (**b**) The distribution of the entire CGMD trajectory before clipping time series in (**a**). (**c**) The distribution of the KL_SAS-CLIP_ for the total of 19,315 time series. They were clipped from the same CGMD trajectories as the #A-1, #A-2, and #A-3. Each satisfies χ^2^ < 3.0. The colors show the appearance probability of the KL_SAS-CLIP_ for each L_t_.

First, the criteria for a clipped time series to be consistent with CGMD simulations were examined in more detail. When L_t_ is too small, the conformational distribution of ER-60 in the clipped time series can be quite different from that of its original trajectory. To visualize this, several time series satisfying χ^2^ < 3.0 were clipped from the same original trajectory as #A-2. The conformational distributions of the time series with L_t_ ≤ 200 ns were clearly sparse and hardly reproduced the distribution of their original 5000-ns trajectory (Fig. 3a, b). Therefore, these conformational distributions did not reproduce the distribution that naturally arises from the CGMD force field. We do not regard such a time series as reflecting a force field. Therefore, we should establish criteria to eliminate the time series. The difference in conformational distribution between a clipped time series and its original trajectory can be a measure of the relationship between the distribution and CGMD force field. Based on this idea, we defined KL_SAS-CLIP_, indicating the difference in (θ_a-b-b′_, θ_b-b′-a′_) probability distribution between a clipped time series and its original trajectory (detailed definition is described in “Methods” section). As expected, KL_SAS-CLIP_s were larger for the time series with smaller L_t_ (Fig. 3a). To examine the relationship between KL_SAS-CLIP_ and L_t_, time series with χ^2^ < 3.0 were extensively collected from the same original trajectories as #A-1, #A-2, and #A-3. In Fig. 3c, a monotonically decreasing curve is clearly observed. The slope of this curve decreased as L_t_ increased, and KL_SAS-CLIP_ remained at ~ 0.5 for the region where L_t_ > 700 ns. Consequently, two conditions “KL_SAS-CLIP_ is approximately 0.5 or less” and “L_t_ is larger than 700 ns” can be criteria for identifying a clipped time series that reflects the CGMD potential function well. Note that #A-1, #A-2, and #A-3 satisfy both the conditions (Table 1). The Kullback–Leibler divergence-based evaluation would be generally applied to other molecules or systems.

Second, the criterion for reproducing the experimental SAXS profile, which was initially χ^2^ < 3.0, was reconsidered. Although the #A-1, #A-2, and #A-3 satisfy the criterion, the squared residuals $\left( {\frac{{{\text{I}}_{{{\text{exp}}}} \left( {\text{Q}} \right) - {\text{I}}_{{{\text{sim}}}} \left( {\text{Q}} \right)}}{{{\upsigma }\left( {\text{Q}} \right)}}} \right)^{2}$ exceeded 20.0 in the region 0.1 Å^−1^ ≤ Q ≤ 0.2 Å^−1^ (Fig. 2c, and residuals are shown in Supplementary Fig. S7a). This Q corresponds to correlation length between 31.4 Å and 62.8 Å. Considering that each of the **a**, **b**, **b′**, and **a′** domains is a globular structure with a diameter of 20–30 Å, the I(Q) of this Q contains information on domain conformation. That is, the SAS-CLIP could extract structural series with incorrect domain conformation even with small χ^2^. According to the observations, we improved the criteria: satisfying both “χ^2^ < 3.0” and “the $\left( {\frac{{{\text{I}}_{{{\text{exp}}}} \left( {\text{Q}} \right) - {\text{I}}_{{{\text{sim}}}} \left( {\text{Q}} \right)}}{{{\upsigma }\left( {\text{Q}} \right)}}} \right)^{2}$ < 12.5 for Q < 0.25 Å^−1^”.

### ***A condition for obtaining time series with large L***_***t***_

To enumerate the possible structural ensembles of ER-60, it is important to efficiently collect ensembles with a large L_t_. We focused on the relationship between λ_WP_ and L_t_. When λ_WP_ was small, L_t_ of the clipped time series tended to be small (Supplementary Fig. S8). Therefore, it was preferable to perform simulations with λ_WP_ ≥ 1.03.

### Enumerating possible ensemble of ER-60 with SAS-CLIP

**Figure 4:**
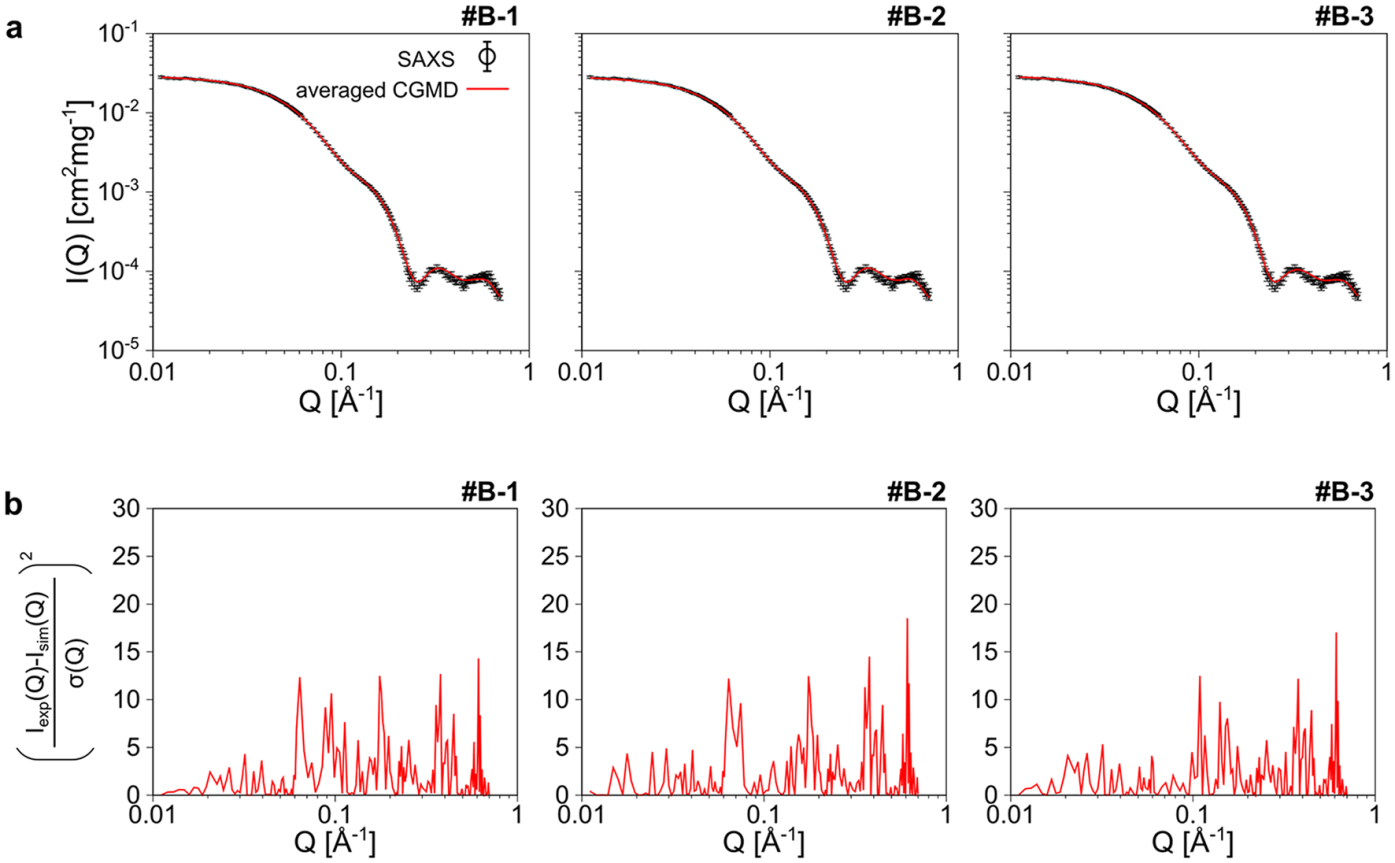
Time series of the SAS-CLIP with the improved criteria. The calculated SAXS profiles of three time series with long L_t_ are shown. (**a**) Averaged SAXS profiles of the time series #B-1, #B-2 #B-3 (red lines) are compared with the experimental ones (black circle with error bars of s.d.). (**b**) The squared residuals of the averaged scattering intensities are shown.

The question whether SAS-CLIP can enumerate various ensembles of ER-60 was addressed. According to the results above, we additionally performed CGMD simulations with λ_WP_ between 1.03 and 1.06 and increased available trajectories. The SAS-CLIP was re-executed with the improved criteria of both “χ^2^ < 3.0” and “$\left( {\frac{{{\text{I}}_{{{\text{exp}}}} \left( {\text{Q}} \right) - {\text{I}}_{{{\text{sim}}}} \left( {\text{Q}} \right)}}{{{\upsigma }\left( {\text{Q}} \right)}}} \right)^{2}$ < 12.5 for Q < 0.25 Å^−1^”. We obtained six time series with L_t_ > 700 ns. In particular, the L_t_ was greater than 1 μs for each of the six time series (Table 1), which were designated as #B-1, #B-2, #B-3, #B-4, #B-5, and #B-6, respectively. KL_SAS-CLIP_ values were reasonably small (Table 1). Their calculated SAXS profile reproduced the experimental one quite well; The new criteria reduced not only the squared residuals (Fig. 4 and Supplementary Fig. S9, and the residuals are presented in Supplementary Fig. S7b) but also the χ^2^ values (Table 1). We noted that the extracted time series included many individual snapshots with large χ^2^ even with the stricter criteria (Supplementary Fig. S10).

**Figure 5:**
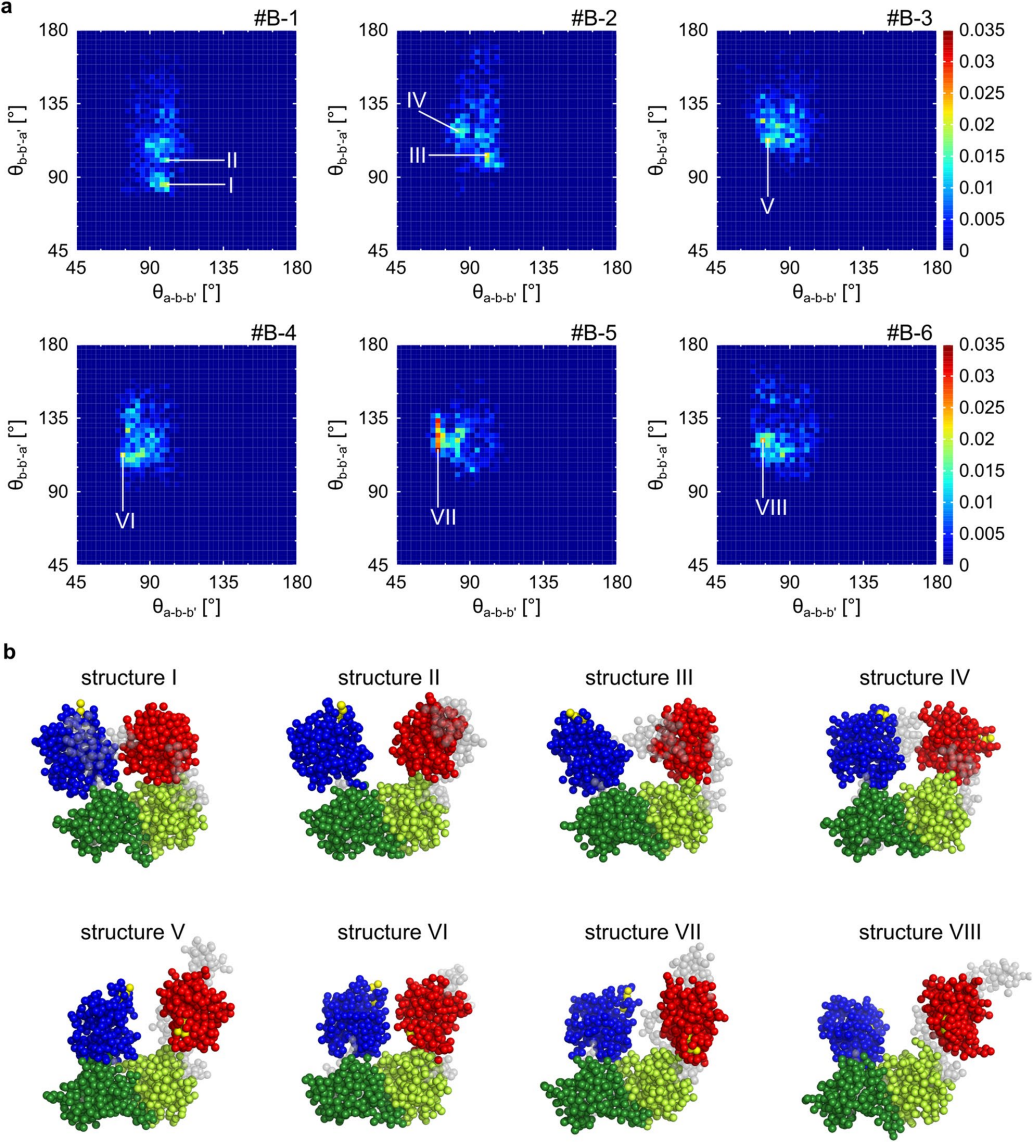
Structure of ER-60 in the clipped time series. (**a**) Domain conformations of ER-60 in the time series #B-1, #B-2, #B-3, #B-4, #B-5, and #B-6 are shown. The color in heatmaps show the appearance probability in the two-dimensional space. (**b**) Structures of ER-60 correspond to the I–VIII. The **a** domain is shown in blue, the **b** domain in green, the **b′** domain in yellowish green, and the **a′** domain in red. The other parts are gray. The reactive cysteines are yellow.

Next, the domain structures obtained with the SAS-CLIP were examined. First, to overview the architecture of ER-60, the two angles θ_a-b-b′_ and θ_b-b′-a′_ were plotted (Fig. 5a). Among the six time series from #B-1 to #B-6, the θ_a-b-b′_ was mainly distributed ranging from 70° to 110° and θ_b-b′-a′_ ranged from 75° to 150°. The distributions were similar for the other time series that satisfied L_t_ > 400 ns (Supplementary Fig. S11 and Table S1). The distribution in Fig. 5a could be roughly classified into two groups. In the first group, the θ_a-b-b′_ distributed at approximately 90° and several clusters were observed in the two-dimensional map (#B-1 and #B-2). In the second group, θ_a-b-b′_ was distributed at < 90° (#B-3, #B-4, #B-5, #B-6). While the structural trends were common, the detailed distributions differed from each other. Additionally, we examined the dihedral angle φ_a-b-b′-a′_. φ_a-b-b′-a′_ distributed between − 15° and 75° (Supplementary Figs. S12, S13). We could not find a clear correlation between φ_a-b-b′-a′_ and θ_a-b-b′_, and between φ_a-b-b′-a′_ and θ_b-b′-a′_.

Subsequently, we examined whether the **a** and **a′** domains prefer a particular orientation in their motion. Supplementary Fig. S14 displays distributions of φ_b′-b-a-CGHC(a)_ and φ_b-b′-a′-CGHC(a′)_ for the six time series. In terms of the domain orientations, one or two preferred ones were observed for each time series. The preferred orientations also differed between the time series. In other words, the SAS-CLIP enumerated the possible distributions of the orientations of the **a**- and **a′-**domains.

Finally, the tertiary structures of ER-60 in the clipped ensembles were overviewed. Several structures featuring θ_a-b-b′_ and θ_b-b′-a′_ with high appearance probability are shown in Fig. 5b. All structures are U-shaped. In structures III and IV, the flexible C-terminal region (Glu^483^-Leu^505^) bridged **a** and **a′** domain. These were temporary bridging in the #B-2. Similar bridging was observed in #B-1, #B-3, #B-4, and #B-5, with lifetimes varying in the range 40–160 ns. The C-terminal loop might contribute to the functional motion of ER-60 via the domain bridging.

In summary, we confirmed that SAS-CLIP can enumerate a variety of ensembles of ER-60. The four-domain architecture could be roughly classified into two classes, whereas the detailed conformational distribution differed among the clipped ensembles. The orientations of the **a**- and **a′-**domains were more diverse among the clipped ensembles.

### Analysis of isosbestic points in the set of scattering curves

**Figure 6:**
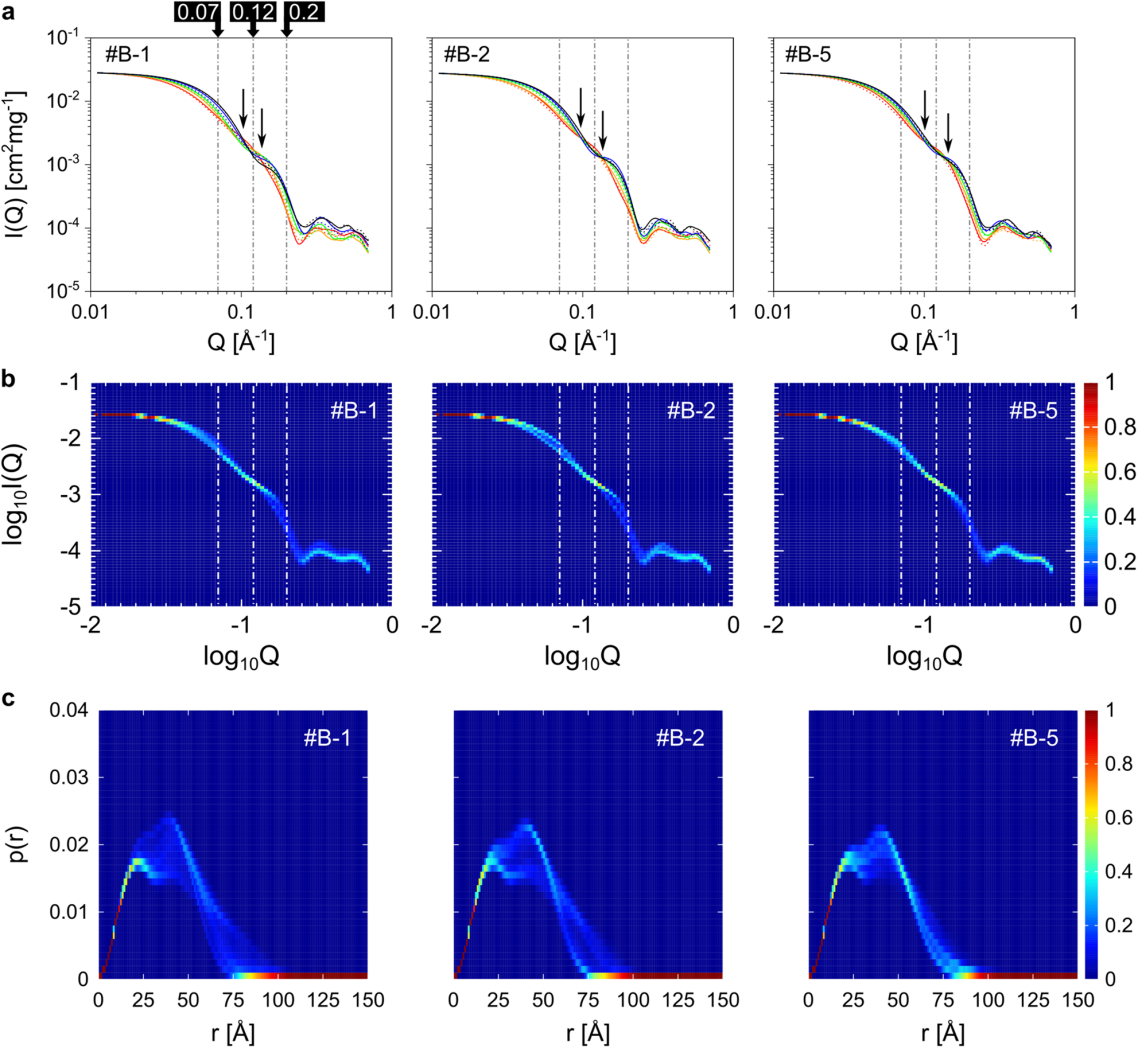
Variety of calculated SAXS profiles in each clipped time series. (**a**) Calculated SAXS profiles of each ER-60 structure. Ten profiles are shown for each time series. Five of them are shown by the red, orange, green, blue, and black dotted lines, respectively. The other five profiles are shown by the solid red, orange, green, blue, and black lines, respectively. For each graph, two approximate isosbestic points are shown by arrows. (**b**) Distributions of I(Q)s over all structures in each time series. The distribution is calculated for each value of log_10_Q. Therefore, for each log_10_Q, sum of the probabilities of log_10_I(Q) is 1. The color shows the appearance probability of log_10_I(Q) values at each log_10_Q. (**c**) Distribution of p(r) for each value of r. Distributions of p(r)s over all structures in each time series are shown. For each r, sum of the appearance probabilities of p(r) is 1. The color shows the appearance probability of p(r) at each r. Here, data are shown for the #B-1, #B-2, and #B-5. Data for the other time series are shown in the Supplementary Figure S15.

Reflecting the structural diversity in each clipped ensemble, their calculated scattering profiles were also diverse (Fig. 6, Supplementary Fig. S15). We next discussed significance of isosbestic points appearing in the set of scattering curves. According to a previous study^40^, an isosbestic point suggests existence of a variable having a approximate linear relationship with the scattering intensity as follows;

$$ {\text{I}}\left( {{\text{Q}},{\text{X}}} \right)\sim {\text{I}}\left( {{\text{Q}},{\text{X}}_{0} } \right) + \left( {{\text{X}} - {\text{X}}_{0} } \right)\frac{{\partial {\text{I}}\left( {{\text{Q}},{\text{X}}} \right)}}{{\partial {\text{X}}}} $$

Here, X is the variable and X_0_ is a parameter. The difference in I(Q) between molecular structures corresponds to difference in the X. Therefore, the X is a conformational coordinate.

In other words, a single molecular motion along the X can explain diversity of calculated SAXS profiles in a structural ensemble when an isosbestic point is found in Q-I(Q) plot. We verified this linear relationship for the ensembles of ER-60 obtained with SAS-CLIP. In Fig. 6a, the calculated scattering curves of 10 structures in the #B-1, #B-2, and #B-5 are shown, respactively. The I(Q) varied with the structure, especially around Q = 0.07 Å^−1^ and Q = 0.2 Å^−1^. In addition, intersections of the curves were concentrated at two regions around Q = 0.12 Å^−1^ (shown by arrows in Fig. 6a). Distribution of I(Q) at each Q value was also calculated to get an overview of all the scattering curves (Fig. 6b). Again, sharp I(Q) distributions were observed around Q = 0.12 Å^−1^. These results indicated that there were two approximate isosbestic points around Q = 0.12 Å^−1^. Corresponding to the I(Q) distribution, p(r) showed sharp distribution between 50 and 60 Å (Fig. 6c). Similary, two isosbestic points were also observed in each of #B-3, #B-4, and #B-6 (Supplementary Fig. S15). The two isosbestic points were not Q values at which only intra-domain scattering appear (Supplementary Fig. S16). Based on the results, a single conformational coordinate was expected to explain the diversity of the SAXS profiles.

**Figure 7:**
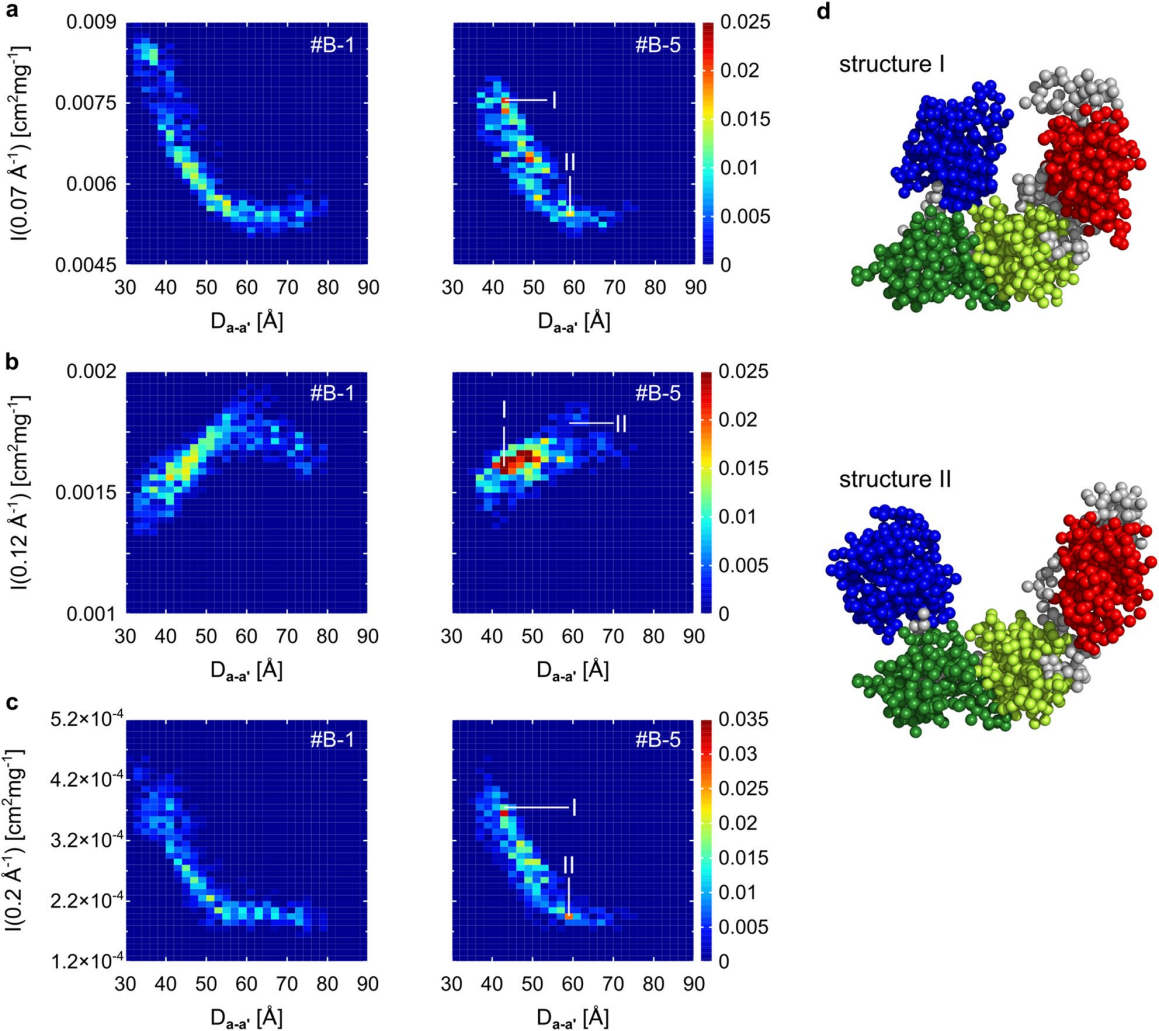
Relationship between I(Q) and domain conformation. **a**–**c** Correlation between the distance D_**a**-**a′**_ and scattering intensity. (**a**) Appearance probabilities of pairs of values the D_**a**-**a′**_ and I(0.07 Å^−1^). The color shows the probability. (**b**) The probabilities of pairs of values the D_**a**-**a′**_ and I(0.12 Å^−1^). (**c**) The probabilities of pairs of values the D_**a**-**a′**_ and I(0.2 Å^−1^). Here, the heatmaps for the time series #B-1 and #B-5 are shown. (**d**) Two typical structures in the #B-5. The **a** domain in shown in blue, the **b** domain in green, the **b′** domain in yellowish green, and the **a′** domain in red. The other parts are in gray. In panels **a**, **b**, and **c**, the pixels to which the structure I and II belong are marked.

Indeed, the distance between centers of **a** and **a′** domain (D_**a-a′**_) approximately linearly correlated with I(Q) (Fig. 7). Here, the relationship between I(Q) and the D_**a-a′**_ was shown for three points, Q = 0.07 Å^−1^, 0.12 Å^−1^, and 0.2 Å^−1^, respectively. Although the linearity depending on the time series (e.g. The relation was clear in #B-5, but that was relatively weak in #B-1.), the relationship was common to the six ensembles (Supplementary Figs. S17, S18, S19).

In summary, our result supports that a single molecular motion can explain diversity of I(Q)s in a structural ensemble when an isosbestic point is found in Q-I(Q) plot. In ER-60, that was open-close motion of the **a** and **a′** domains.

### Interpretation and application of SAS-CLIP

SAS-CLIP does not provide a unique solution of the structural ensemble of a biomolecule. The structural ensemble obtained by this method differ from each other, but they all reproduce the same SAXS profile. This also means that any combination of these time series also reproduce the experimental SAXS profile. Additionally, the structural distribution of each clipped time series approximates that of a long-term MD simulation. Therefore, each time series obtained by SAS-CLIP can be an element of motion of a biomolecule, and any combination of these are candidates for the real structural ensemble.

Here, we propose a method to identify a structural ensemble of a biomolecule using clipped time series and another experimental data. With **X** as a quantity obtained in an experiment other than SAXS, **X** will be expressed as follows:

$$ {\mathbf{X}} = \mathop \sum \limits_{{{\text{i}} = 1}}^{{\text{N}}} {\text{c}}_{{\text{i}}} {\mathbf{X}}_{{{\text{SAS}} - {\text{CLIP}},{\text{ i}}}} $$

where N is the number of structural series obtained from SAS-CLIP. The **X**_SAS-CLIP,i_ is the quantity for i-th structure series of the SAS-CLIP, which is averaged over all structures in the i-th series. c_i_ is a weight factor. A reasonable structural ensemble that is consistent with any of SAXS, CGMD, and another experiment can be obtained by simply determining the c_i_s. We can assume a variety of data are the quantity **X**. In fact, many experimental data represent the average quantity over all molecules in solution. In addition, many kinds of experimental data can be calculated from a given atomistic structures. For example, the profile of small-angle neutron scattering (SANS)^41–44^, chemical shift for nuclear magnetic resonance (NMR)^45,46^, and efficiency of fluorescence resonance energy transfer (FRET)^47^ can be calculated from tertiary structures. The proposed method has at least two advantages. First, the method is advantageous for constructing realistic ensembles of highly flexible biomolecules; Combination of clipped ensembles with SAS-CLIP results in an ensemble with a very large number of structures. Second, resultant ensembles are consistent with a force field of MD simulation regardless of c_i_ values. The linear combination approach can also avoid the possible effect of artificial free energy minimum derived from an incorrect simulation force field, which is often a problem in the entropy maximization approach. Sufficient ensembles should be clipped to perform such an analysis.

SAS-CLIP can be applied to atomistic MD simulations. It is easier for the atomistic simulations to compare a simulation step with real time. When the SAS-CLIP is applied to atomistic simulations, extracted time series will be useful to analyze experimental data including temporal information, such as neutron spin echo.

When L_t_ is large, KL_SAS-CLIP_ is sufficiently small (Table 1). If we regard an original CGMD trajectory as a subspace of the free energy landscape, each clipped time series reproduces “a subspace of the subspace”. In other words, the SAS-CLIP provides time series that roughly trace a subspace of a free energy landscape of a biomolecule. This contrasts with the entropy maximization approach, where an entire free energy landscape is reproduced as much as possible. We designed our method not to narrow down the behavior of a biomolecule based on two facts. First, an SAXS profile does not contain enough information to identify structural ensemble of a biomolecule with high resolution. Second, simulation force fields contain incorrectness. Instead of narrowing an ensemble down, SAS-CLIP is designed to be easily combined with other experimental data.

In practice, there are three major advantages of SAS-CLIP. First, time series can be extracted from relatively short MD simulations with low computational cost. We can obtain many time series at the same time when multiple simulations are performed in parallel. Second, obtained structural sets reflect a force field of MD simulations, and thus they are candidates of “element of motions”. Third, this method makes it easy to obtain a structural ensemble which matches SAXS, MD, and another experiment with simple linear model. This method will provide a new way to study biomolecules by integrating various type of experiments.

## Supplementary Information

Supplementary Information.

## Data Availability

The datasets generated during and/or analysed during the current study are available from the corresponding author on reasonable request.

## Code availability

GROMACS: https://www.gromacs.org/.

GROMACS 2020.4: https://doi.org/10.5281/zenodo.4054979.

PyMOL: https://pymol.org/2/.

Martini v3 beta: http://cgmartini.nl/index.php/martini3beta.

martinize2: https://github.com/marrink-lab/vermouth-martinize.

Pepsi-SAXS: https://team.inria.fr/nano-d/software/pepsi-saxs/.

Backward: http://cgmartini.nl/index.php/tools2/resolution-transformation.

Gnuplot: http://www.gnuplot.info/.

Inkscape: https://inkscape.org/.
