# Supplementary material for: Extracting time series matching a small-angle X-ray scattering profile from trajectories of molecular dynamics simulations
Source: Sci Rep. 2022 Jun 15;12:9970. doi: 10.1038/s41598-022-13982-9 (PMC9200744; doi:10.1038/s41598-022-13982-9)
Supplement: Supplementary file 1 — Supplementary Information. [file 41598_2022_13982_MOESM1_ESM.pdf]

SUPPLEMENTARY INFORMATION

Extracting time series matching a small-angle X-ray scattering profile from trajectories of molecular dynamics simulations

Masahiro Shimizu*, Aya Okuda, Ken Morishima, Rintaro Inoue, Nobuhiro Sato, Yasuhiro Yunoki, Reiko Urade and Masaaki Sugiyama*

Institute for Integrated Radiation and Nuclear Science, Kyoto University, Kumatori, Sennan-gun, Osaka 590-0494, Japan.

*: Correspondence e-mail: shimizu.masahiro.3n@kyoto-u.ac.jp, sugiyama@rri.kyoto-u.ac.jp

Supplementary Tables

Table S1.

λ_{WP}, L_t, χ^2, and $KL_{SAS\text{-}CLIP}$ of other time series obtained by the SAS-CLIP

ID	Criteria	λ_{WP}	L_t [ns]	χ^2	$KL_{SAS\text{-}CLIP}$	
#B-7	I. $\chi^2 < 3.0$	1.06	636	2.86	0.626	same trajectory as #A-3
#B-8	II. squared	1.049	634	2.73	0.648	
#B-9	residuals < 12.5	1.046	598	2.47	0.805	from a 10-μs simulation
#B-10	($Q < 0.25$ Å^{-1})	1.055	568	2.66	0.868	from a 10-μs simulation
#B-11		1.05	498	2.99	0.990	
#B-12		1.049	432	2.61	1.00	

Supplementary Figures

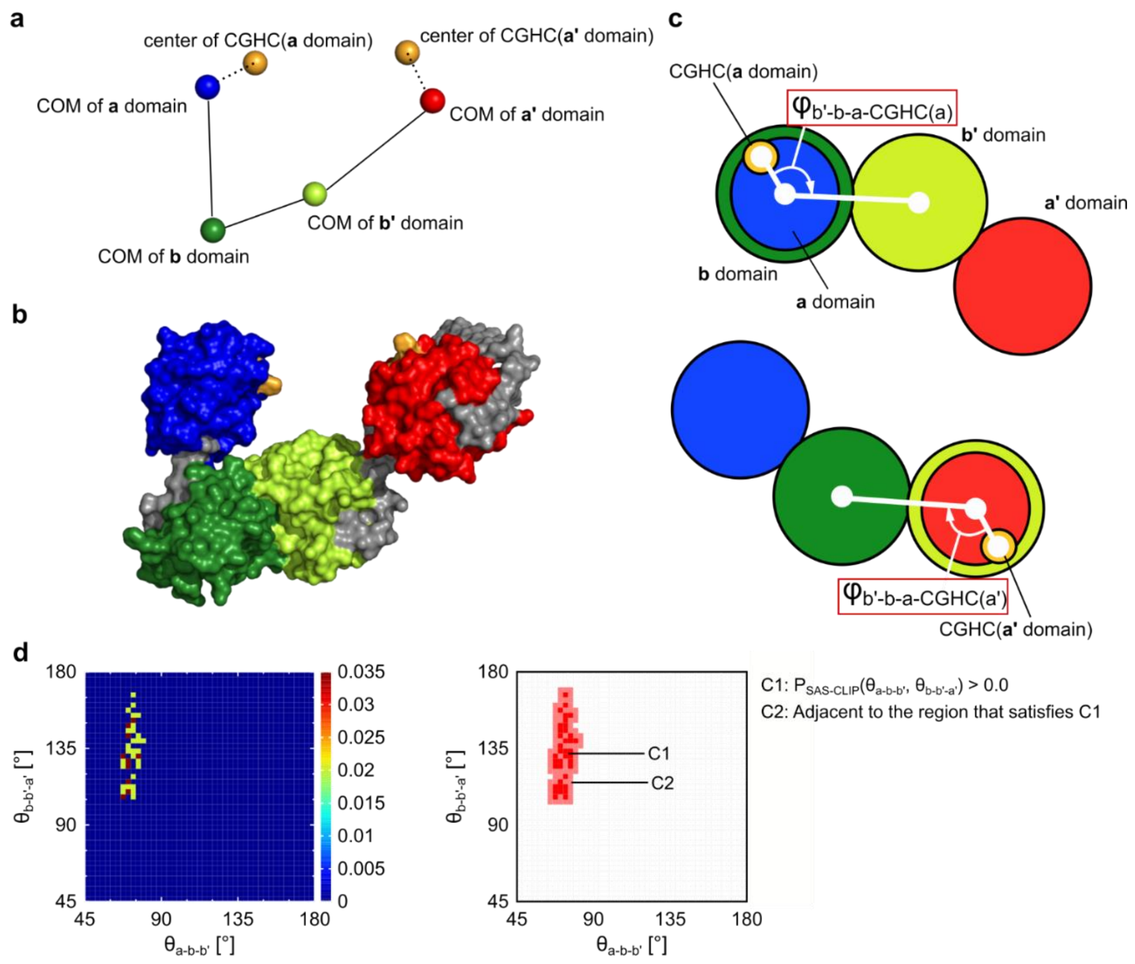

Fig. S1. Description of overall architecture of ER-60.

a. COMs of the **a**, **b**, **b'**, and **a'** domains. COMs of the CGHC motifs in the **a** and **a'** domains are also shown. The definition of these COMs is described in the **Methods**.

b. Atomistic model of ER-60 corresponding to panel **a**.

c. A schematic view of the $\varphi_{b'\text{-}b\text{-}a\text{-}CGHC(a)}$ and $\varphi_{b\text{-}b'\text{-}a'\text{-}CGHC(a')}$. Color code is the same as Fig. 1.

d. Definition of a region in ($\theta_{a\text{-}b\text{-}b'}$, $\theta_{b\text{-}b'\text{-}a'}$)-space which is referred to calculate the $KL_{SAS\text{-}CLIP}$. A conformational distribution is shown in the left, and the corresponding region used to calculate the $KL_{SAS\text{-}CLIP}$ is shown in the right.

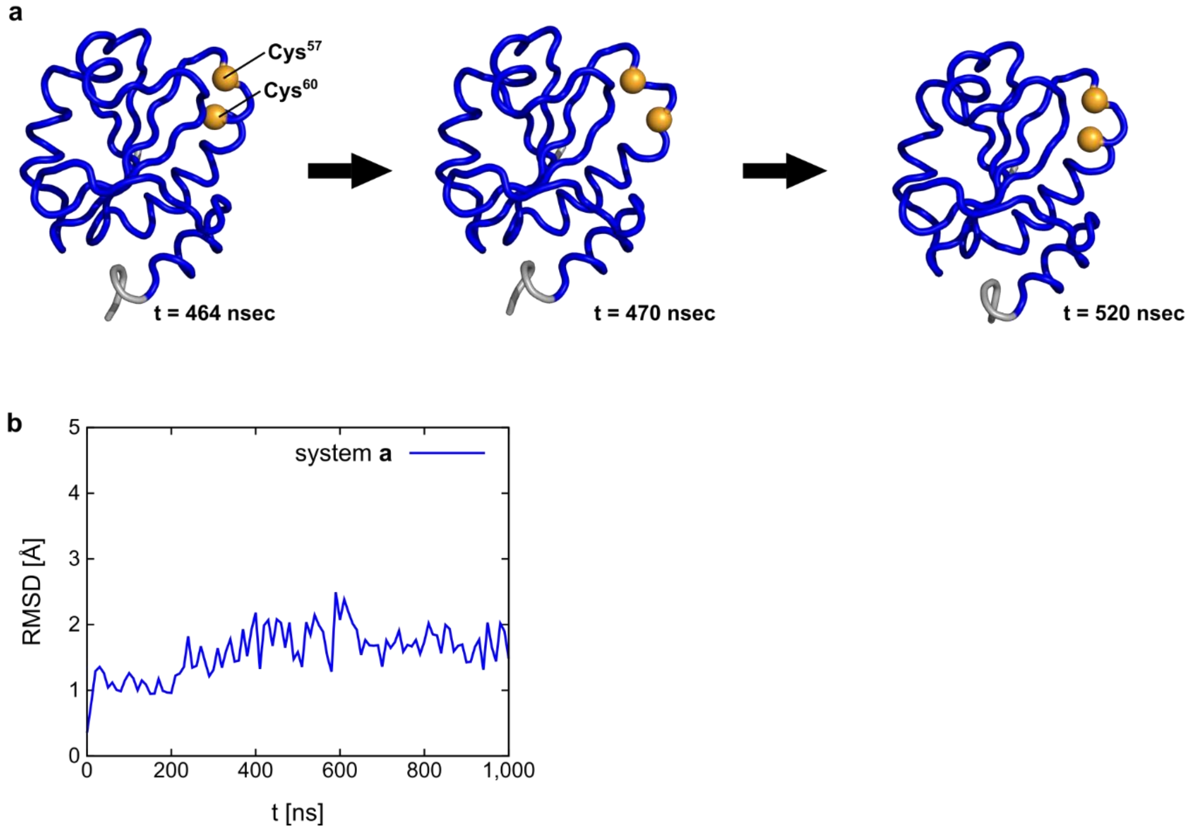

Fig. S2. Spontaneous structural change in CGHC motif of a domain in the atomistic MD simulation.

a. Three structures of **a** domain at t = 464, 470, and 520 ns in the 1 μs atomistic MD simulation. The **a** domain is colored blue. The reactive cysteines are colored orange. **b**. Trajectory of RMSD against the crystal structure.

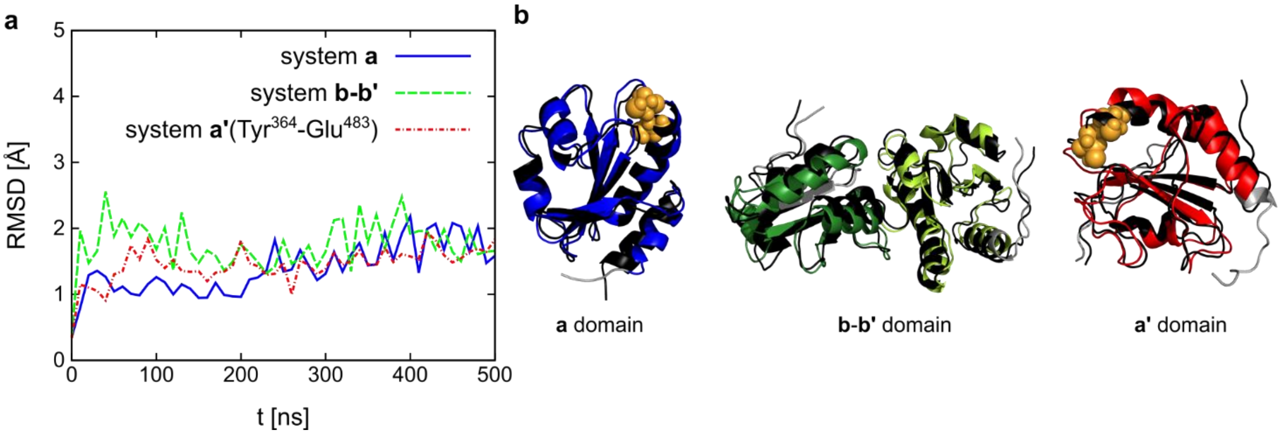

Fig. S3. Structural fluctuation of three ER-60 regions during atomistic MD simulations.

a. Change in RMSD against the crystal structure in each simulation. The RMSDs were calculated for Cα atoms. For the system **a** and the system **b-b'**, RMSDs were calculated for entire region. For the system **a'**, RMSD were calculated for globular region (Tyr^{364} to Glu^{483}). The detailed definition of each system is provided in the Methods.

b. Representative snapshots are superposed on the crystal structure. The color code of the MD snapshots is the same as in Fig. 1. The crystal structure is colored in black.

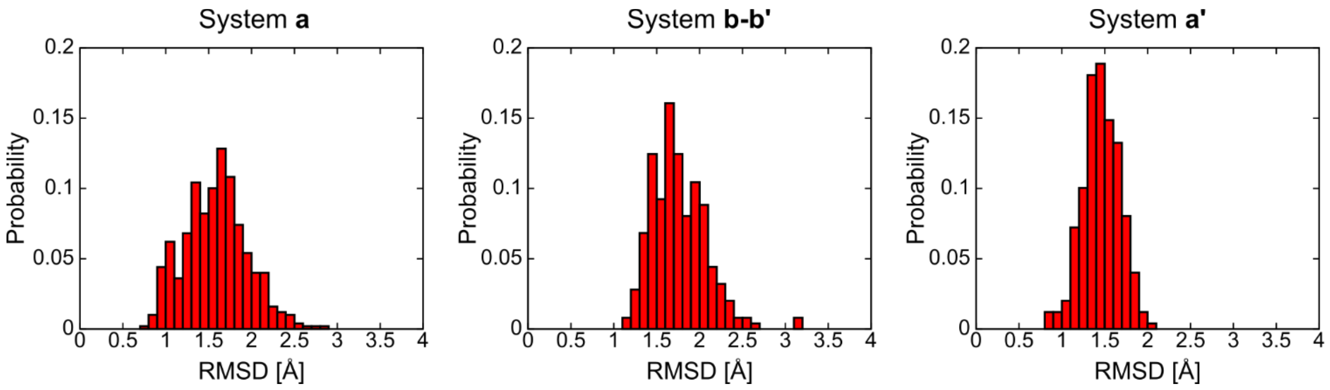

Fig. S4. Distributions of the RMSD of individual atomistic MD snapshots.

Distributions are shown for each of the three MD trajectories. System **a** includes the **a** domain. System **b-b'** includes the **b** and **b'** domain. System **a'** includes the **a'** domain.

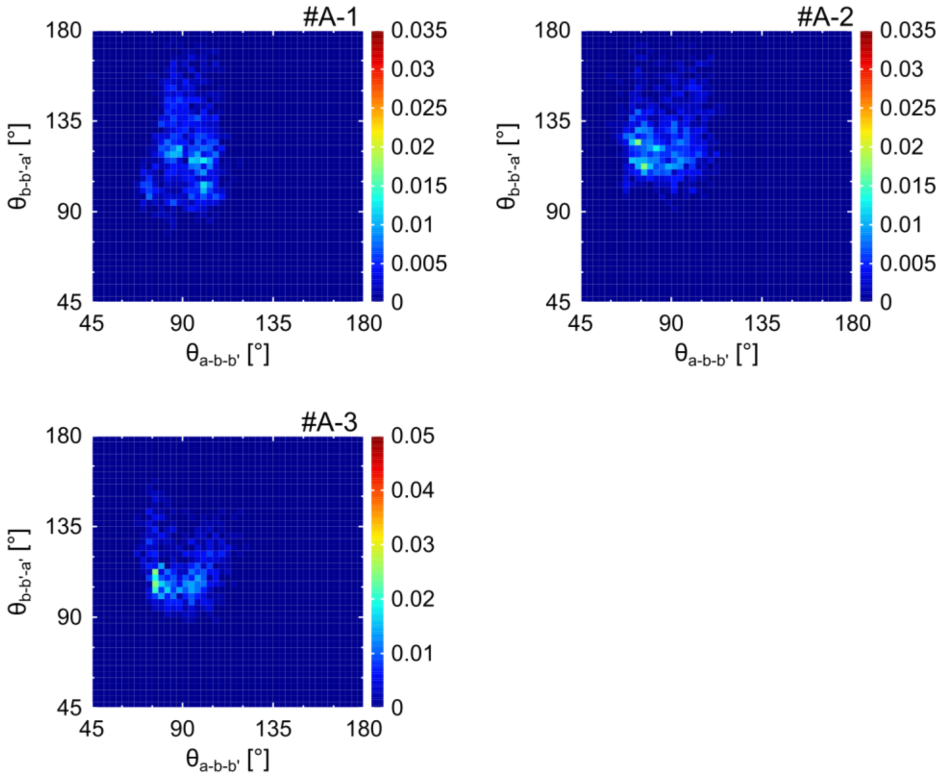

Fig. S5. Conformational distributions of ER-60 in the time series #A-1, #A-2, and #A-3.

Distributions of domain conformation of ER-60 in the time series #A-1, #A-2, and #A-3 are shown.

The color in heatmaps shows appearance probability in the ($\theta_{a\text{-}b\text{-}b'}$, $\theta_{b\text{-}b'\text{-}a'}$)-space.

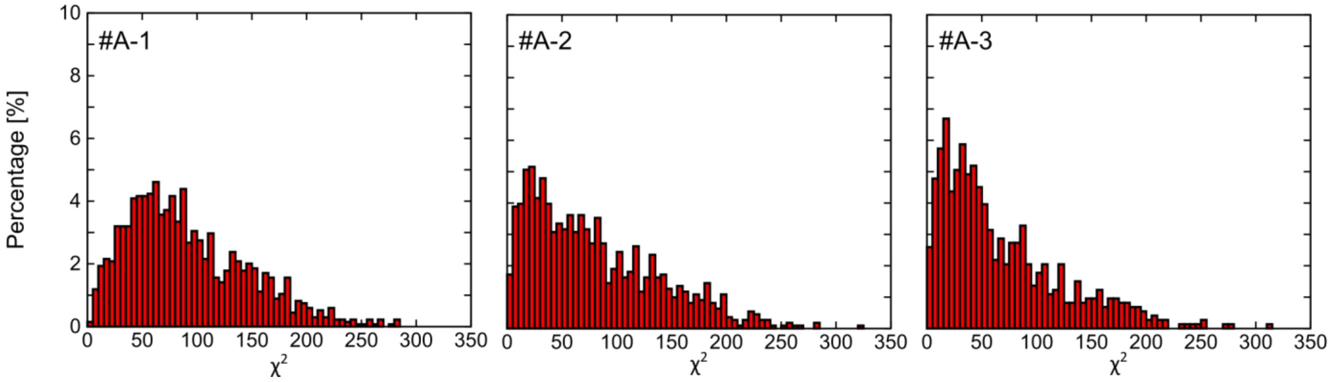

Fig. S6. Distributions of χ^2 value of individual CGMD snapshots

Distribution histogram is shown for the time series #A-1, #A-2, and #A3.

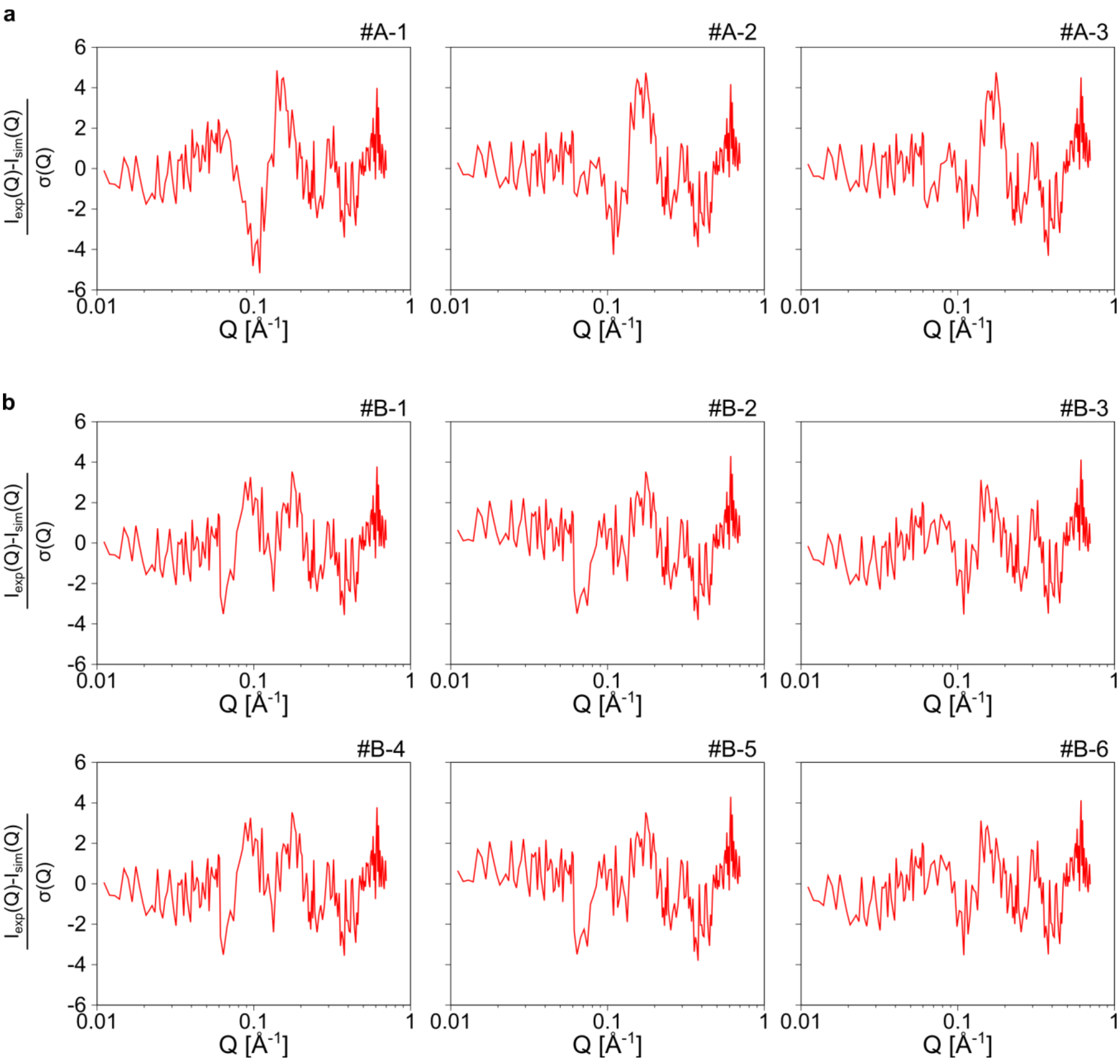

Fig. S7. The residuals $\left(\frac{I_{exp}(Q)-I_{sim}(Q)}{\sigma(Q)}\right)$ of the averaged scattering intensities.

a. Residuals of time series clipped with the criterion $\chi^2 < 3.0$.

b. Residuals of time series clipped with the criteria of both $\chi^2 < 3.0$ and $\left(\frac{I_{exp}(Q)-I_{sim}(Q)}{\sigma(Q)}\right)^2 < 12.5$ (for Q < 0.25).

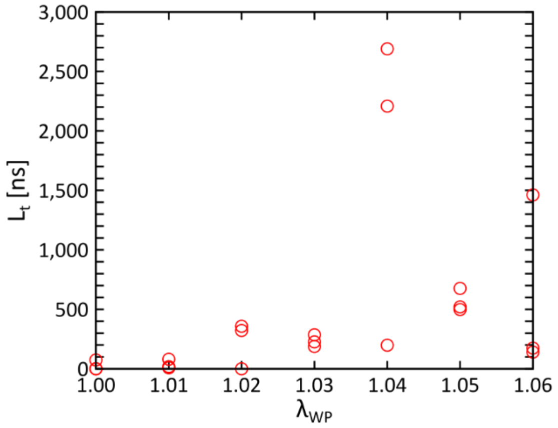

Fig. S8. Relationship between the λ_{WP} and the L_t.

The L_t for each clipped time series is shown by a red circle. Each time series satisfies $\chi^2 < 3.0$.

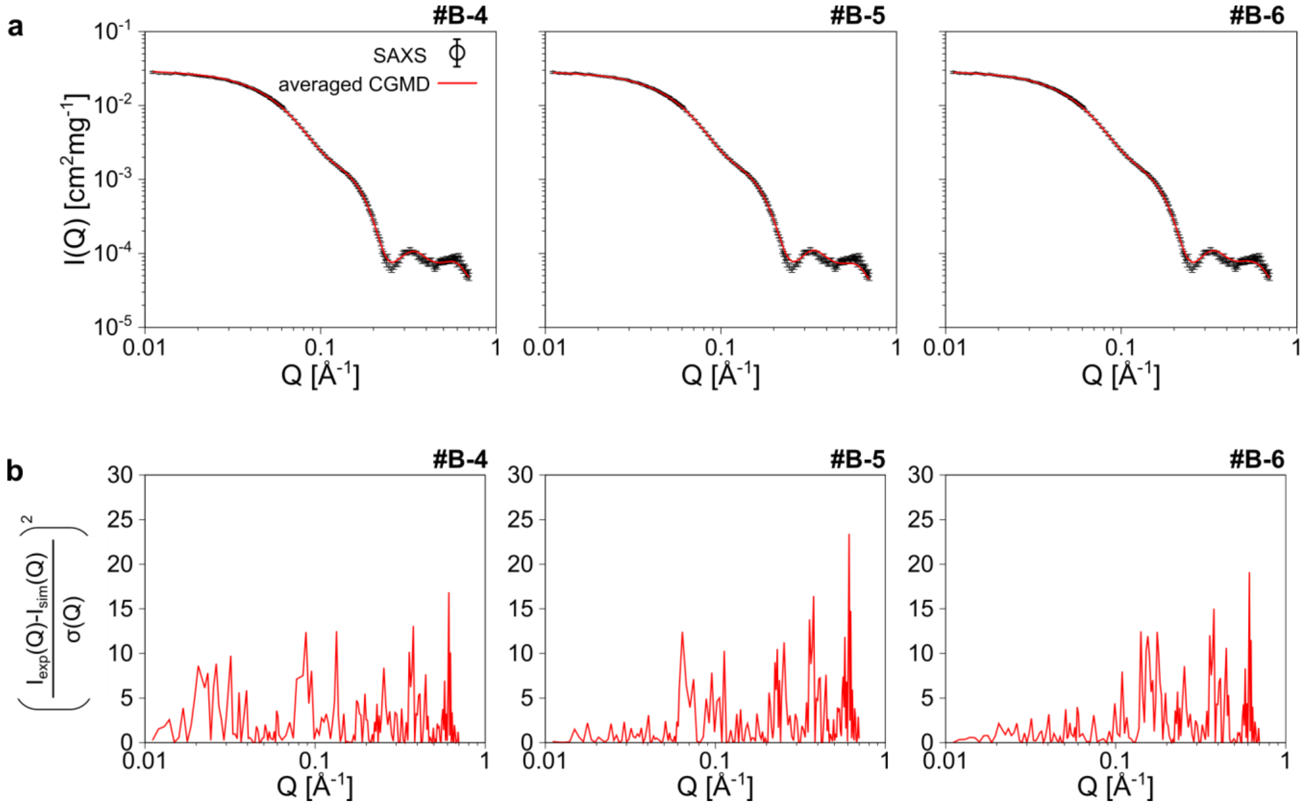

Fig. S9. Time series of the SAS-CLIP with the improved criteria.

Calculated SAXS profiles of other three time series with long L_t are shown.

a. Averaged SAXS profiles of the time series #B-4, #B-5, and #B-6 (red lines) are compared with the experimental profiles (black circle; error bars demote standard deviation).

b. Squared residuals of the averaged scattering intensities.

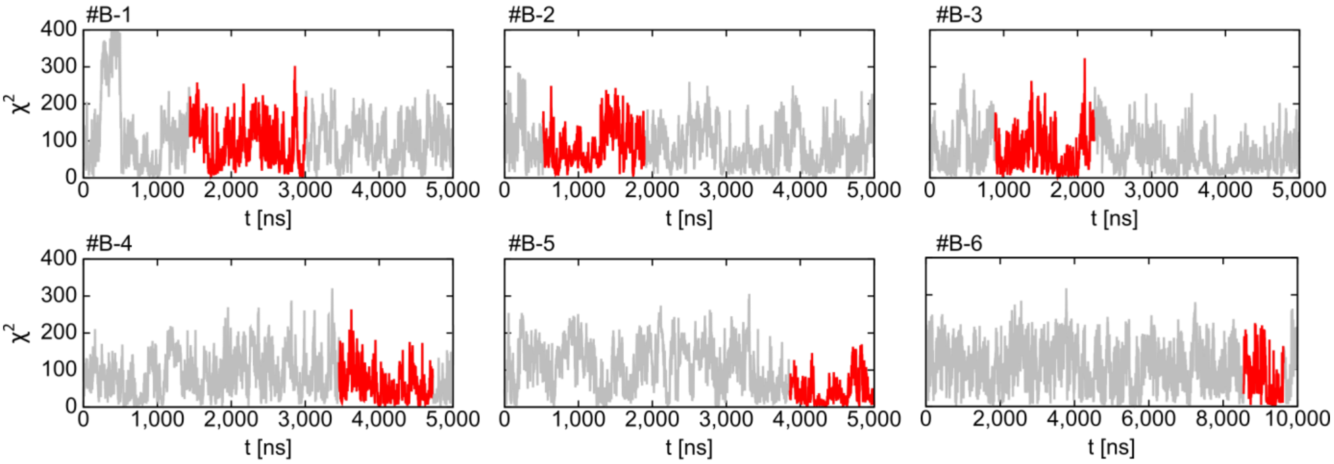

Fig. S10. Trajectory of χ^2 of the time series.

Trajectories of χ^2 during CGMD simulations. The clipped time series #B-1, #B-2, #B-3, #B-4, #B-5, and #B-6 are shown in red. The other regions are shown in gray.

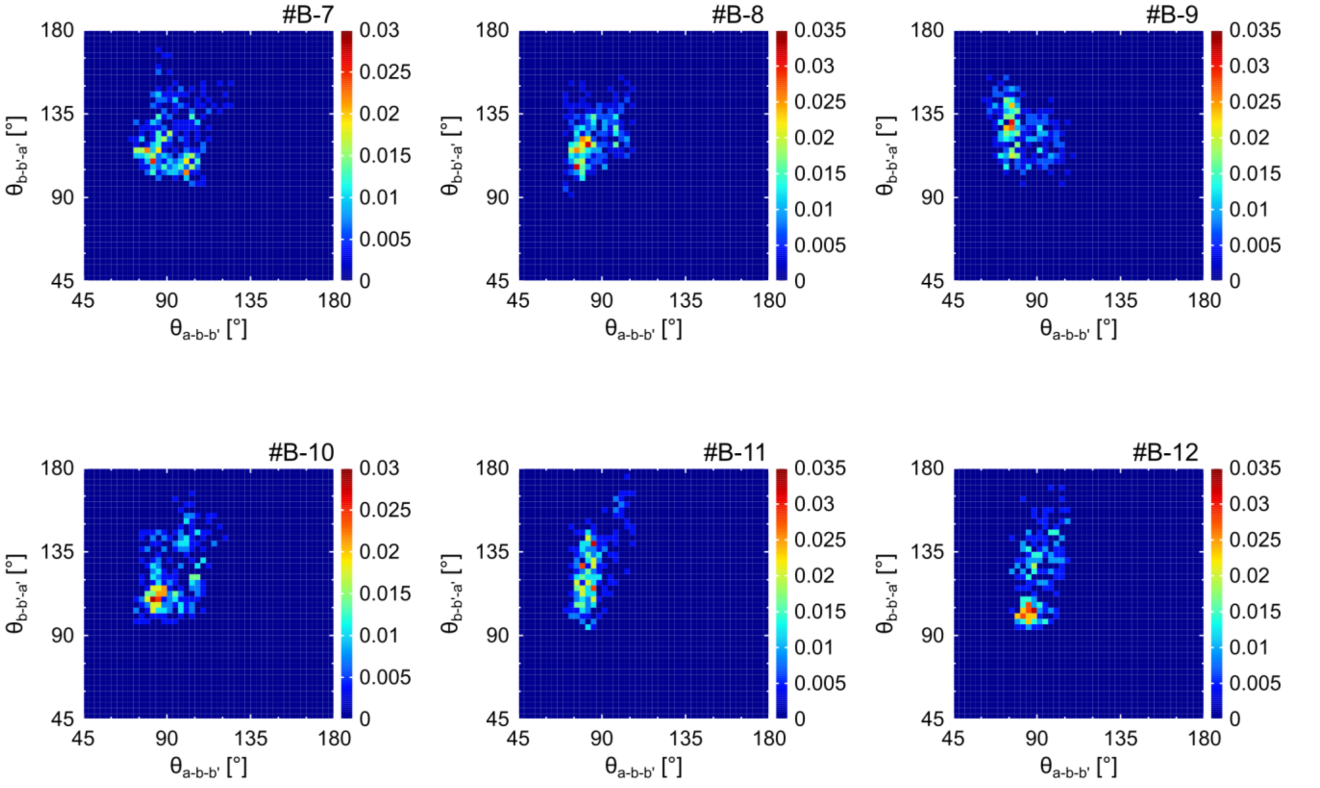

Fig. S11. Conformation of ER-60 in extracted time series.

Domain conformations of other time series (from #B-7 to #B-12) are shown. In these time series, the L_ts are small and the $KL_{SAS\text{-}CLIP}$s are large (See Table. S1). The color shows the appearance probability in the ($\theta_{a\text{-}b\text{-}b'}$, $\theta_{b\text{-}b'\text{-}a'}$)-space.

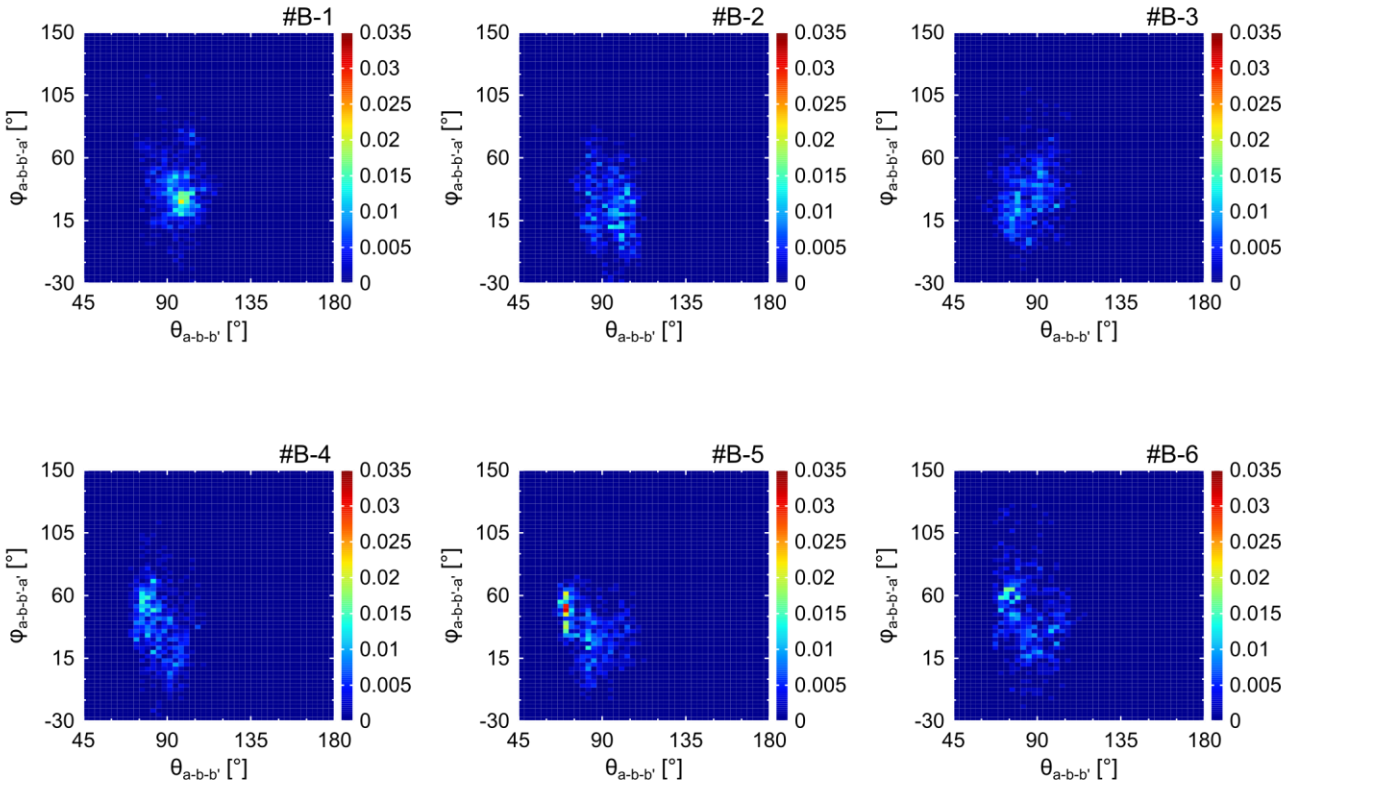

Fig. S12. Distribution of $\theta_{a\text{-}b\text{-}b'}$ and $\varphi_{a\text{-}b\text{-}b'\text{-}a'}$ in the clipped time series.

Distributions are shown for the six structural series, from #B-1 to #B-6. The color shows the appearance probability in the ($\theta_{a\text{-}b\text{-}b'}$, $\varphi_{a\text{-}b\text{-}b'\text{-}a'}$)-space.

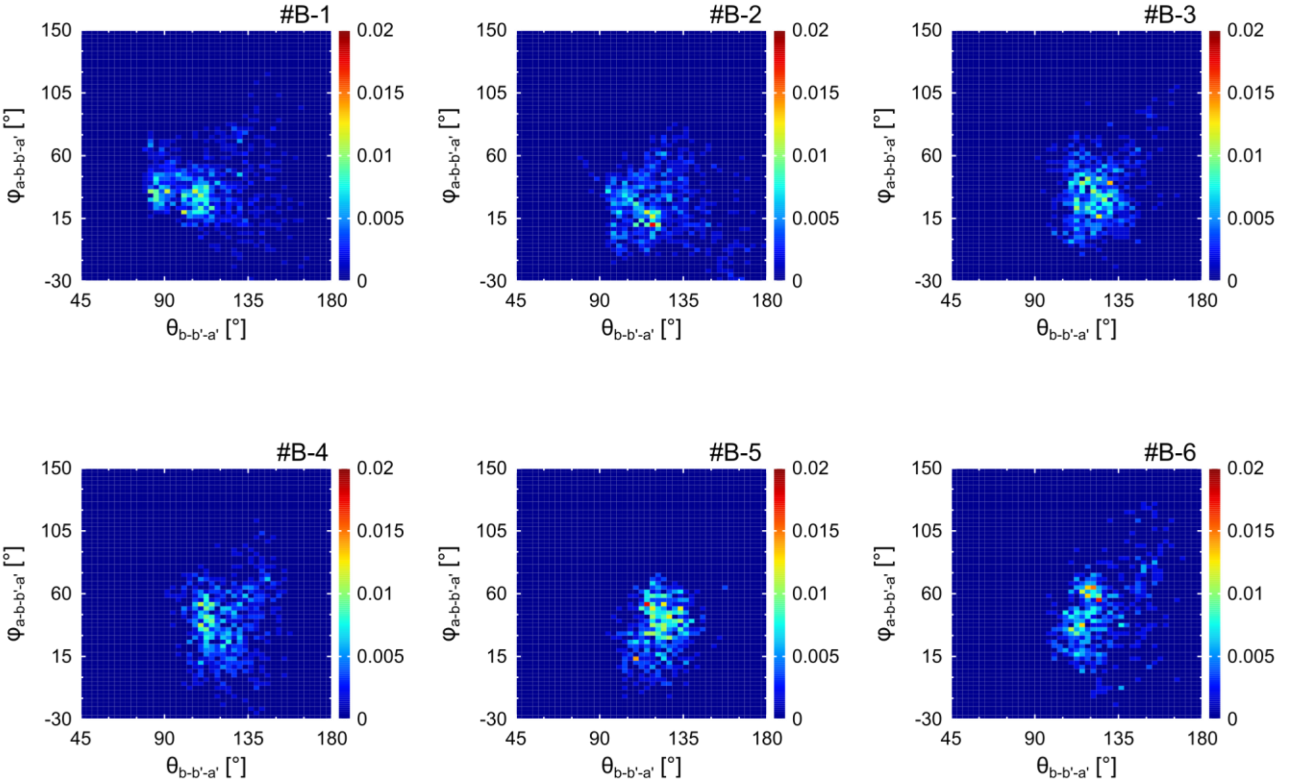

Fig. S13. Relationship between $\theta_{b\text{-}b'\text{-}a'}$ and $\varphi_{a\text{-}b\text{-}b'\text{-}a'}$.

Distributions are shown for the six structural series. The color shows the appearance probability in the ($\theta_{b\text{-}b'\text{-}a'}$, $\varphi_{a\text{-}b\text{-}b'\text{-}a'}$)-space.

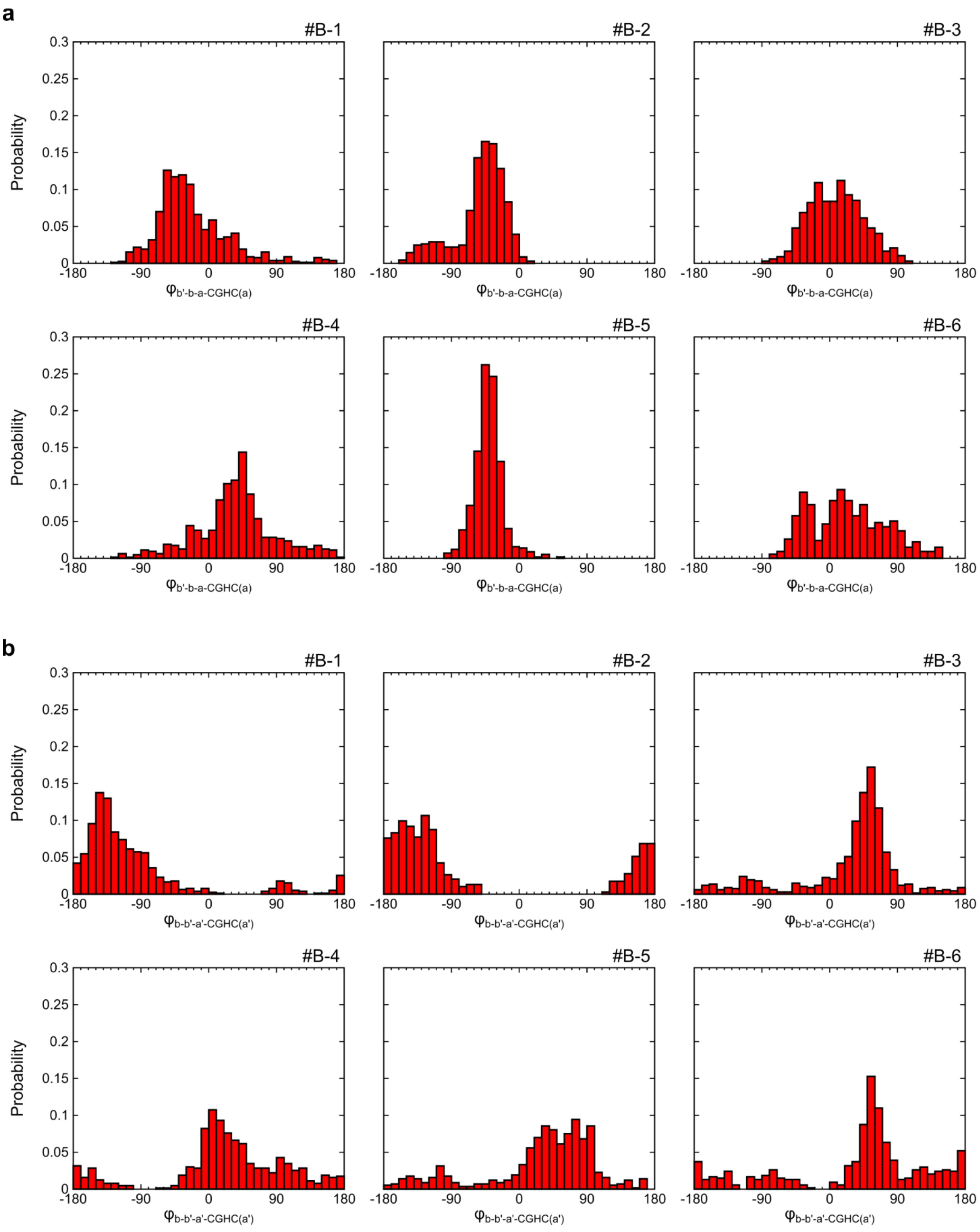

Fig. S14. Orientation of CGHC motifs in the a and the a' domains.

Distributions of $\varphi_{b'\text{-}b\text{-}a\text{-}CGHC(a)}$ (**a**) and $\varphi_{b\text{-}b'\text{-}a'\text{-}CGHC(a')}$ (**b**).

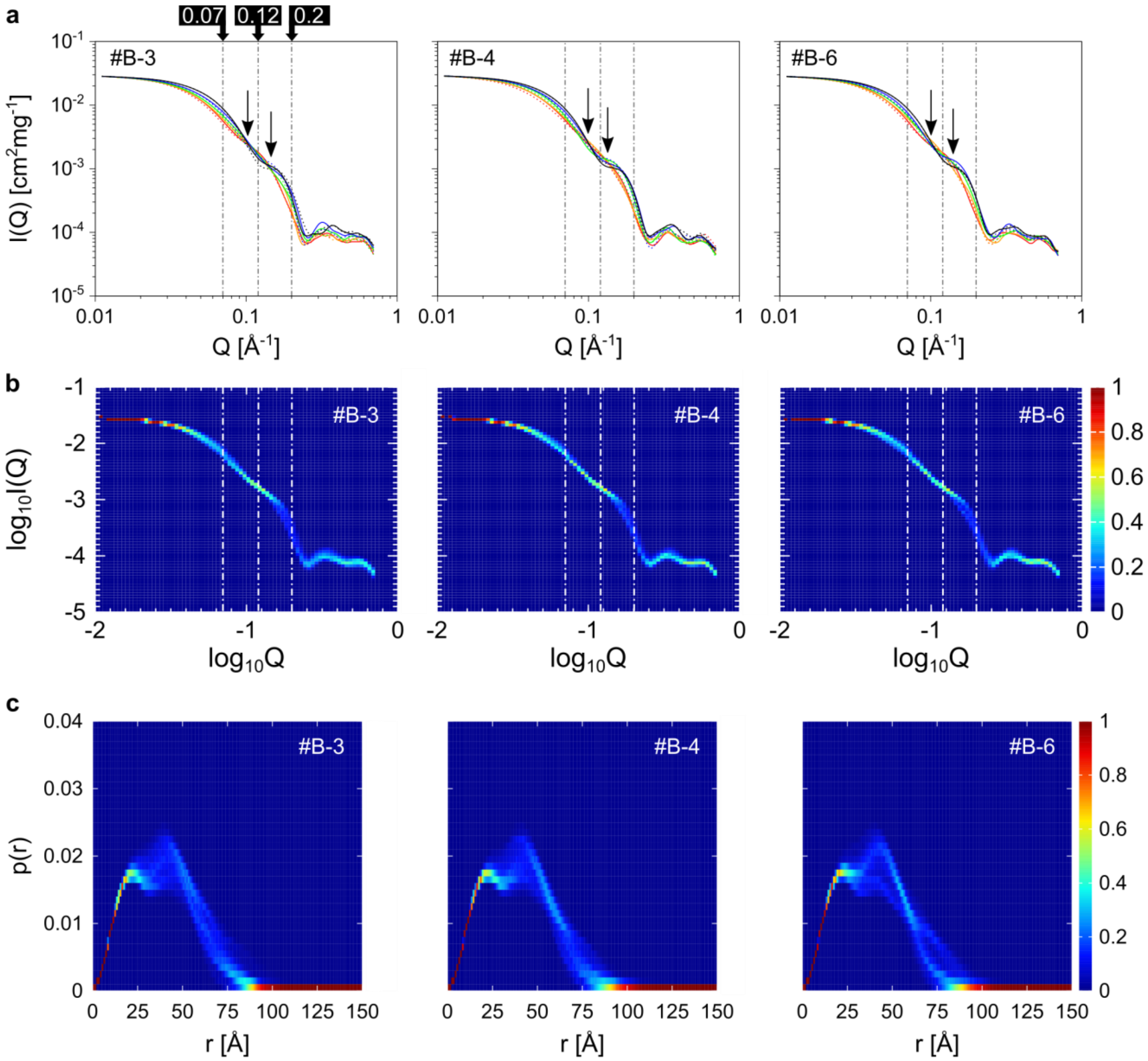

Fig. S15. Variety of calculated SAXS profiles in each clipped time series.

a. Calculated SAXS profiles of each ER-60 structure. Ten profiles are shown for each time series. Five of them are shown by the red, orange, green, blue, and black dotted lines, respectively. The other five profiles are shown by the solid red, orange, green, blue, and black lines, respectively. For each graph, two approximate isosbestic points are shown by arrows. **b.** Distributions of I(Q)s over all structures in each time series. The distribution is calculated for each value of $\log_{10}Q$. Therefore, for each $\log_{10}Q$, sum of the probabilities of $\log_{10}I(Q)$ is 1. The color shows the appearance probability of $\log_{10}I(Q)$ at each

$\log_{10}Q$. **c.** Distribution of p(r) for each value of r. Distributions of p(r)s over all structures in each time series are shown. For each r, sum of the appearance probabilities of p(r) values is 1. The color shows the appearance probability of p(r) at each r. Here, data are shown for the #B-3, #B-4, and #B-6.

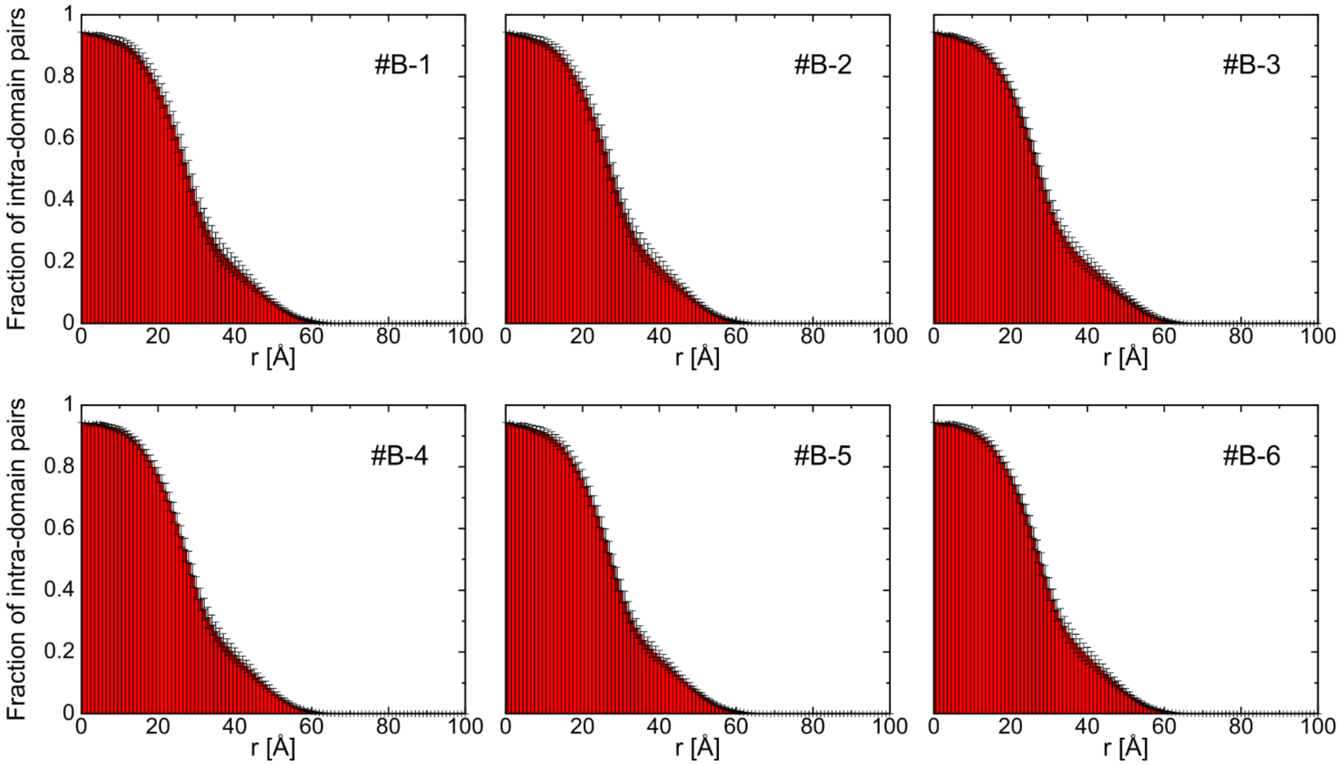

Fig. S16. Fraction of intra-domain electron pairs to all electron pairs.

The histograms are calculated for each r. The error bars represent standard deviations for the all structures in each time series. The fractions are approximated; All electrons were calculated as being in the center of an atom, and hydrogen atoms were ignored. The isosbestic points discussed in this study correspond to r values between 50 and 60 Å.

Here, "an intra-domain electron pair" is defined as an electron pair satisfying one of the following conditions; I. an electron pair within the region Ser^{25}-Gln^{131}, II. an electron pair within the region Ala^{135}-Arg^{363}, or III. an electron pair within the region Ser^{367}-Glu^{483}. This definition is based on the elastic network potential in CGMD simulations.

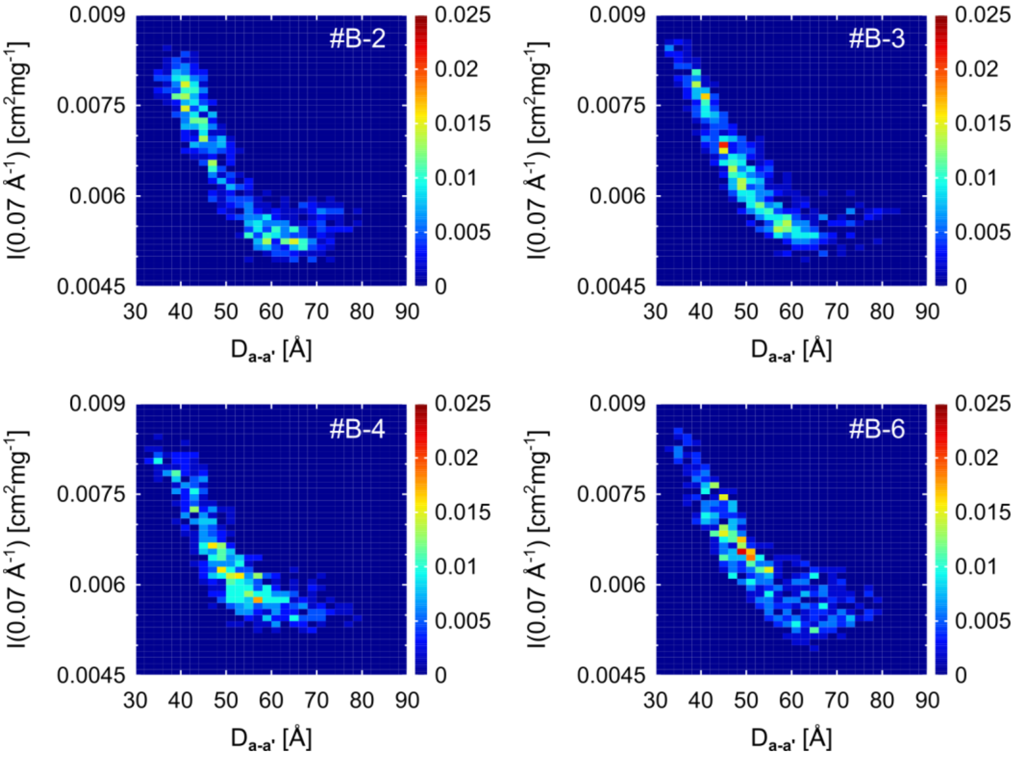

Fig. S17. Relationship between the distance $D_{a\text{-}a'}$ and scattering intensity at Q = 0.07 Å^{-1}.

Appearance probabilities of pairs of values the $D_{a\text{-}a'}$ and the I(0.07 Å^{-1}) are shown. The color of each bin shows the probability. Here, probability heatmaps of #B-2, #B-3, #B-4, and #B-6 are shown.

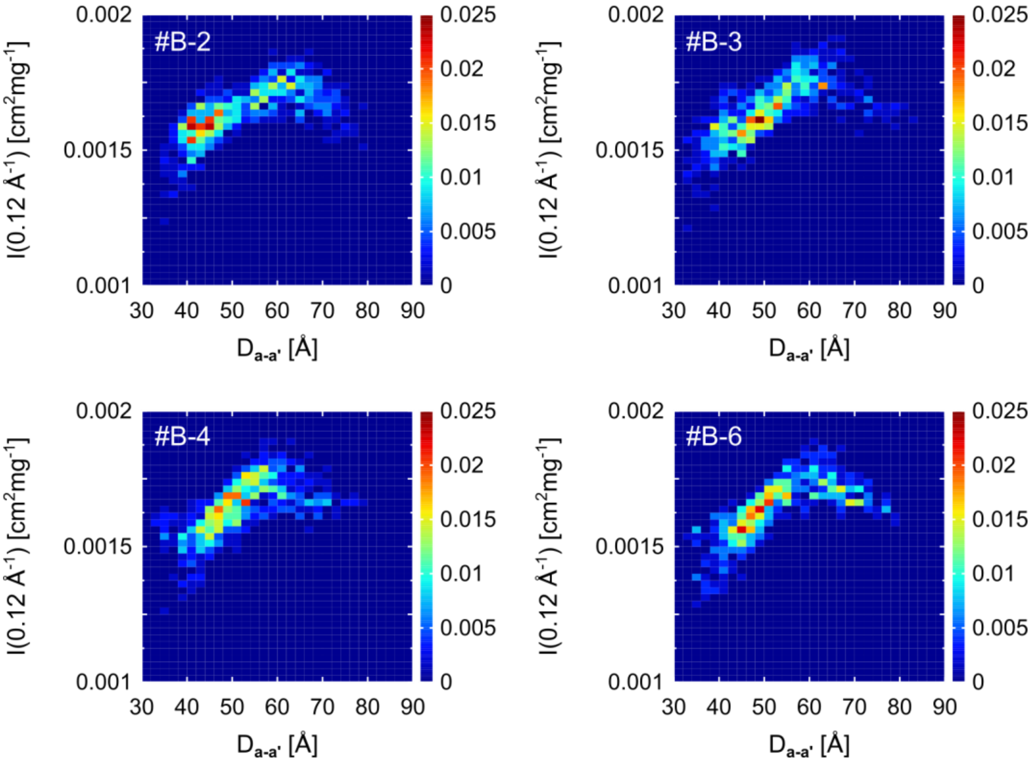

Fig. S18. Relationship between the distance $D_{a\text{-}a'}$ and scattering intensity at Q = 0.12 Å^{-1}.

Appearance probabilities of pairs of values the $D_{a\text{-}a'}$ and the I(0.12 Å^{-1}) are shown. The color of each bin shows the probability. Here, probability heatmaps of #B-2, #B-3, #B-4, and #B-6 are shown.

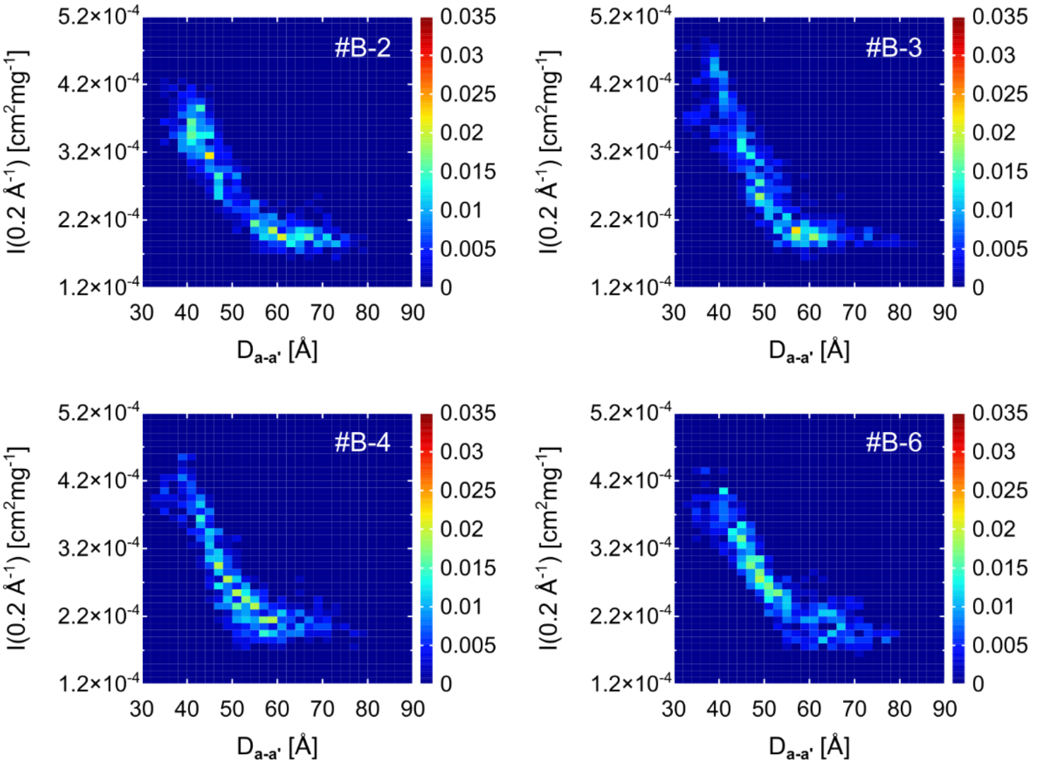

Fig. S19. Relationship between the distance $D_{a\text{-}a'}$ and scattering intensity at Q = 0.2 Å^{-1}.

Appearance probabilities of pairs of values the $D_{a\text{-}a'}$ and the I(0.2 Å^{-1}) are shown. The color of each bin shows the probability. Here, probability heatmaps of #B-2, #B-3, #B-4, and #B-6 are shown.

Supplementary Methods

Detail of atomistic MD simulations

The initial structures were prepared based on the crystal structure of ER-60 C60A mutant (PDBID: 3F8U)[1] with two modifications. First, the mutated residue was changed to cysteine using PyMOL[2]. Second, a C-terminal loop (from Lys^{482} to Glu^{493}) was changed to an extended conformation bound to the **a'** domain in the crystal structure. The simulations were performed in cubic boxes. The simulation box of the system **a** contains the ER-60 fragment, 11,444 water molecules, 36 Na^{+} ions, and 33 Cl^{-} ions. The simulation box of the system **b-b'** contains the ER-60 fragment, 28,677 water molecules, 87 Na^{+} ions, and 83 Cl^{-} ions. The simulation box of the system **a'** contains the ER-60 fragment, 28,634 water molecules, 86 Na^{+} ions, and 80 Cl^{-} ions. The Amber ff14SB force field and TIP3P water model[3,4] were employed. The electrostatic interactions were calculated with the particle-mesh Ewald method[5]. The production runs were preceded by energy minimization by the steepest descent algorithm, equilibration under NVT condition at 300 K for 100 ps, and equilibration under NPT condition at 300K, with 1 bar for 1 ns. During the equilibrations, the coordinates of all heavy atoms were restrained to their initial positions. For the systems **b-b'** and **a'**, the production runs were performed under NPT condition at 300K and 1 bar for 500 ns. For the system **a**, the production run was performed under NPT condition at 300K and 1 bar for 1,000 ns because of the large conformational change in the CGHC motif of **a** domain just before t = 500 ns (Fig. S2). In the simulations, except for the energy minimization, all bonds with hydrogen atoms in protein were constrained by LINCS, and water molecules are constrained by SETTLE[6,7]. The NPT condition was realized by the velocity rescaling method developed by Bussi et al[8] with $\tau_t = 0.1$ ps, and the Parrinello-Rahman's pressure coupling algorithm[9] ($\tau_p = 2.0$ ps).

Detail of coarse-grained (CG) MD simulations

CGMD simulations were performed using Martini 3 open-beta version[10]. The simulation system contains a single ER-60 molecule, 44,531 water beads, 537 Na^+ ions, and 527 Cl^- ions.

To maintain the folded structure, an elastic network potential is also defined between backbone beads that are more than three amino acids apart in the sequence. The residue pair to define the elastic network potential was determined based on the atomistic MD simulations as follows. First, each snapshot of the atomistic MD simulations was mapped to the corresponding CG model. Second, the elastic network potential was defined for amino acid pairs satisfying the following conditions: average distance of their backbone-bead pair < 9.0 Å and standard deviation of the distance < 0.5 Å. These calculations were done for the last 90% of each trajectory to avoid possible bias of the initial structure. We noted that fluctuation of the ER-60 fragment at their terminus may differ from that of the full-length ER-60. The elastic network potential of amino acids related to Gln^{131}, Ala^{132}, Gly^{133}, Pro^{134}, Tyr^{364}, Leu^{365}, and Lys^{366} were defined based on the crystal structure. In the latter case, the elastic network was defined when a distance between two backbone beads was < 9.0 Å. The force constant of the elastic network model was 500 $kJmol^{-1}nm^{-2}$ as described previously[11].

The initial structure of CGMD simulations was prepared by connecting representative structures of the **a**, **b-b'**, and **a'** domains in corresponding atomistic simulations. Each representative structure was selected as a structure that gives the smallest elastic-network potential energy. Finally, the C-terminal region was generated by PyMOL.

The topology file of CGMD simulations was generated by Martinize2[12]. The scfix option was utilized to fix sidechains[13]. To ensure the flexibility of hinge between folded regions and the C-terminal loop, scfix was turned off for Pro^{134}, Tyr^{364}, Leu^{365}, Lys^{366}, and the C-terminal loop.

The production runs were preceded by two relaxation steps. First, energy minimization by the steepest descent algorithm was performed. Second, equilibration under NPT condition was performed at

300K and 1 bar for 1 ns with velocity-rescaling temperature coupling algorithm and Berendsen's pressure coupling algorithm[14]. In equilibrium runs, coordinates of all backbone beads were restrained. Production runs were performed under NPT condition at 300K and 1 bar. In the production runs, the temperature was controlled by the velocity rescaling method developed by Bussi et al with $\tau_t = 1.0$ ps and the pressure was controlled by the Parrinello-Rahman's pressure coupling algorithm with $\tau_p = 12.0$ ps. First, we performed three 5,000-ns production runs with each λ_{WP} of 1.0, 1.01, 1.02, 1.03, 1.04, 1.05, and 1.06. Based on the results, we additionally performed two 5,000-ns production runs with each λ_{WP} of 1.035, 1.043, 1.045, 1.046, 1.049, 1.052, and 1.055. In addition, we performed two 10,000-ns production runs with each λ_{WP} of 1.04, 1.043, 1.046, 1.049, 1.052, and 1.055. Snapshots taken every 2 ns were used for analysis.

Detail of reconstruction of atomistic models.

First, we used the software backward[15] and reverse-mapped all simulation snapshots. However, there were snapshots where backward calculations did not converge. For them, atomistic structures were reconstructed using the other atomistic models already obtained with the backward. The reconstruction procedure was as follows. This was done after performing backward for all snapshots.

[Preparation 1]

We defined a structure pool. This pool included pairs of a CG model and corresponding atomistic model obtained by the backward. The members of the pool were restricted to snapshots belonged to the same simulation trajectory as we wanted to reverse-map. The reason for the restriction is to reduce computation time.

[Preparation 2]

Full-length ER-60 was considered as a set of 28 parts. The reverse-mapping was done for each part separately. The 28 parts are composed of four globular parts (Ser^{25}-Ala^{132}, Ala^{135}-Phe^{241}, Gly^{242}-Tyr^{364}, and Lys^{366}-Glu^{483}), two hinge parts (Gly^{133}-Pro^{134} and Leu^{365}), and 22 single amino acids in the C-terminal flexible loop.

[Reverse-mapping]

For a structure we wanted to reverse-map (here we call “target”), the most similar structure was searched for in the structure pool and superposed. The model search and superposition were performed for each of 28 parts separately. The detailed procedure was as follows:

Step1. A model pair (a CG model and corresponding atomistic model) was extracted from the structure pool.

Step2. The extracted model pair was superposed on the target using SC1 and BB particles in the CG model. The region to superpose depended on type of the part.

Globular part

The region to superpose was the same as the part. For example, the SC1 and BB particles in the region Ser^{25}-Ala^{132} were superposed when reverse-map the part Ser^{25}-Ala^{132}.

Leu^{505}

The SC1 and BB particles in Asp^{504}-Leu^{505} were superposed. The reason for using a larger region than that to be reverse-mapped is to reverse-map backbone of flexible loop more precisely.

The other parts

The SC1 and BB particles in the part and its neighboring amino acids were superposed. For example, the SC1 and BB particles in the region Glu^{503}-Leu^{505} were superposed when reverse-map the part Asp^{504}.

Step3. Calculated RMSD for the superposed SC1 and BB particles.

Step4. Performed Step1-3 for all the model pairs in the structure pool.

Step5. A model pair with the smallest RMSD in the pool was regarded as the most suitable model. The coordinates of atoms of the most suitable model after superposition were defined as the reverse-mapped atomistic model. When the region used for superposition was larger than the region to reverse-map, the atoms out of the region to reverse-map were ignored.

Supplementary References

1. Dong, G., Wearsch, P. A., Peaper, D. R., Cresswell, P. & Reinisch, K. M. Insights into MHC class I peptide loading from the structure of the tapasin-ERp57 thiol oxidoreductase heterodimer. *Immunity* **30,** 21-32 (2009).
2. The PyMOL Molecular Graphics System, Version 1.8, Schrödinger, LLC.
3. Maier, J. A. et al. ff14SB: Improving the accuracy of protein side chain and backbone parameters from ff99SB. *J. Chem. Theory Comput.* **11,** 3696-3713 (2015).
4. Jorgensen, W. L., Chandrasekhar, J., Madura, J. D., Impey, R. W. & Klein. M. L. Comparison of simple potential functions for simulating liquid Water. *J. Chem. Phys.* **79,** 926-935 (1983).
5. Essmann, U. et al. A smooth particle mesh Ewald method. *J. Chem. Phys.* **103,** 8577-8593 (1995).
6. Hess, B., Bekker, H., Berendsen, H. J. C. & Fraaije, J. G. E. M. LINCS: A linear constraint solver for molecular simulations. *J. Comput. Chem.* **18,** 1463-1472 (1997).
7. Miyamoto, S. & Kollman, P. A. Settle: An analytical version of the SHAKE and RATTLE algorithms for rigid water models. *J. Comput. Chem.* **13,** 952-962 (1992).
8. Bussi, G., Donadio, D. & Parrinello, M. Canonical sampling through velocity rescaling. *J. Chem. Phys.* **126,** 014101 (2007).
9. Parrinello, M. & Rahman, A. Polymorphic transitions in single crystals: A new molecular dynamics method. *J. Appl. Phys.* **52,** 7182-7190 (1981).
10. Martini v3 beta: http://cgmartini.nl/index.php/martini3beta.
11. Periole, X., Cavalli, M., Marrink, S. J. & Ceruso, M. A. Combining an elastic network with a coarse-grained molecular force field: structure, dynamics, and intermolecular recognition. *J. Chem. Theory Comput.* **5,** 2531-2543 (2009).
12. Martinize2 https://github.com/marrink-lab/vermouth-martinize
13. Herzog, F. A., Braun, L., Schoen, I. & Vogel, V. Improved side chain dynamics in MARTINI

Simulations of protein-lipid interfaces. *J. Chem.Theory Comput.* **12,** 2446-2458 (2016).

14. Berendsen, H. J. C., Postma, J. P. M., van Gunsteren, W. F., DiNola, A. & Haak, J. R. Molecular dynamics with coupling to an external bath. *J. Chem. Phys.* **81,** 3684-3690 (1984).

15. Wassenaar, T. A., Pluhackova, K., Böckmann, R. A., Marrink, S. J. & Tieleman, D. P. Going backward: A Flexible Geometric Approach to Reverse Transformation from Coarse Grained to Atomistic Models. *J. Chem. Theory Comput.* **10,** 676-690 (2014). 10.1021/ct400617g, Pubmed:26580045.
